# What services are currently provided to people with heart failure with preserved ejection fraction in the UK and what are their components? A systematic scoping review

**DOI:** 10.1093/eurjcn/zvaf143

**Published:** 2025-07-17

**Authors:** Faye Forsyth, Christi Deaton, Paul R Kalra, Mark Green, Mary E Harrison, Sara Tavares, Andreas Dirksen, Isla Kuhn, Barbara Farquharson, Rosalynn C Austin

**Affiliations:** Department of Public Health and Primary Care, University of Cambridge, Robinson Way, Cambridge CB2 0SR, UK; KU Leuven Department of Public Health and Primary Care, KU Leuven, Kapucijnenvoer 7, PB7001, Leuven 3000, Belgium; Department of Public Health and Primary Care, University of Cambridge, East Forvie Building, Cambridge CB2 0SR, UK; Department of Cardiology, Portsmouth Hospitals University NHS Trust, Portsmouth PO6 3LY, UK; Department of Cardiology, Portsmouth Hospitals University NHS Trust, Portsmouth PO6 3LY, UK; Department of Medicine, University Hospitals of Leicester NHS Trust, Infirmary Square Leicester LE1 5WW and University of Leicester, University Road, Leicester LE1 7RH, UK; Heart Failure Team, Heart Failure Offices, Ealing Community Cardiology, Imperial College NHS Trust, Praed Street, London W2 1NY, UK; Heart & Vascular Center, University Medicine Frankfurt, Theodor-Stern-Kai 7, Frankfurt am Main 60596, Germany; Medical Library, University of Cambridge, Cambridge CB2 0QQ, UK; Nursing, Midwifery and Allied Health Professional's Research Unit (NMAHP), Centre for Healthcare and Community Research (CHeCR), University of Stirling, Stirling FK9 4NF, UK; Department of Public Health, Faculty of Health Sciences, University of Stavanger, Stavanger 4021, Norway; Department of Cardiology, Portsmouth Hospitals University NHS Trust, Southwick Hill, Portsmouth PO6 3LY, UK; NIHR Applied Research Collaborative (ARC) Wessex, Innovation Centre, Science Park, 2 Venture Rd, Chilworth, Southampton SO16 7NP, UK

**Keywords:** Guidelines, Heart failure, HFpEF, Scoping review

## Abstract

**Aims:**

This study aims to review the clinical services currently provided to people with heart failure with preserved ejection fraction (HFpEF) living in the UK, to examine the format of clinical care, and to assess these against UK guideline recommendations provided by the National Institute for Health and Care Excellence (NICE).

**Methods and results:**

A systematic scoping review was performed. We synthesized articles narratively according to the systematic review without meta-analysis guidelines, drawing on other established recommendations for narrative methods. We critically appraised articles using Critical Appraisal Skills Programme tools. Following screening (*n* = 11 495) and full-text review (*n* = 68), we included 25 unique reports from databases and nine operations documents received following a public appeal. Overall reporting within both published articles and operations documents was sub-optimal, limiting our description of service provision. From the data available, it appeared that most services were NICE guideline compliant for overall heart failure management; however, multiple services augmented clinical teams with additional specialists to assist in the management of people with HFpEF. Thematic analysis suggested *variability* in HFpEF services and *uncertainty* over the optimal clinic format and management strategies, which was complicated by *complexity* in patients. Cumulatively, publications suggested there is a need for service re-design.

**Conclusion:**

Heart failure with preserved ejection fraction care in the UK appears variable, and the format of optimal services to improve outcomes is not yet clear. Patient complexity makes management challenging. Although some clinical services have made efforts to adapt to HFpEF patients’ needs, there remain significant gaps in service provision and care. Findings underscore a pressing need for service re-design.

**Registration:**

This scoping review protocol was published on the public Open Science Framework platform (no registration reference provided) and can be accessed at https://osf.io/5gufq/.

## Graphical Abstract

**Graphical Abstract:**
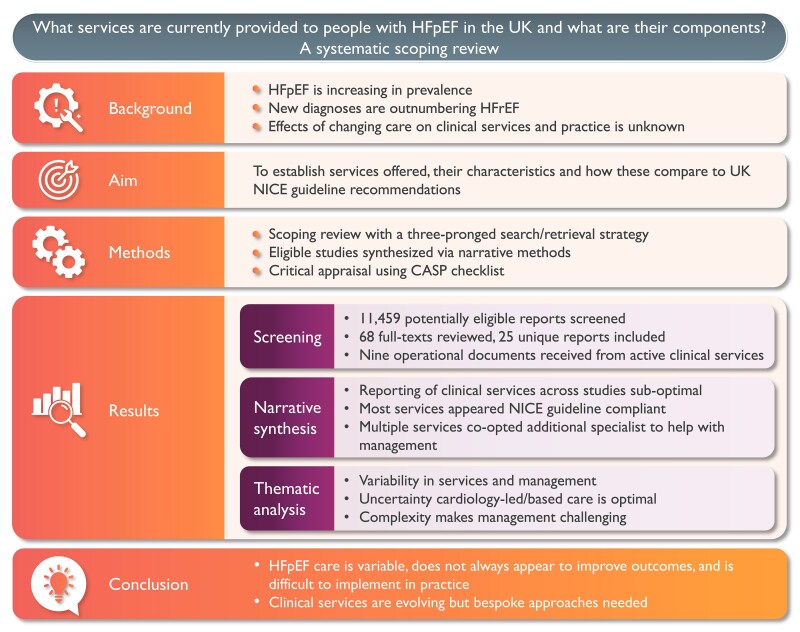

Novelty There have been no published reviews of clinical services in the UK specifically for people with heart failure with preserved ejection fraction (HFpEF). This review of 11 459 reports identified 25 papers that described a clinical service managing HFpEF in the UK and nine operations documents provided by clinically active personnel. Through data synthesis, we were able to identify that:

- Clinical services appear to adhere to the broad heart failure management recommendations set out in the National Institute for Health and Care Excellence (NICE) guidelines.
- Lack of details and clear algorithms for HFpEF within NICE guidelines may have contributed to the variability observed.
- Multiple services had employed additional specialist staff to support them in the management of HFpEF.
- Thematic analysis suggested there is *variability* in service provision, *uncertainty* over the optimal HFpEF model, and difficulty managing HFpEF due to *complexity*.
- Cumulatively, these themes suggest there is a pressing need for service re-design; however, questions remain over the optimal approach.

## Introduction

In the UK, 920 000 people are estimated to have heart failure,^1^ a significant proportion of which will have the phenotype heart failure with preserved ejection fraction (HFpEF). Unfortunately, national statistics on the incidence and prevalence of HFpEF are not available.^2^ However, if we extrapolate from epidemiological data,^3,4^ then an estimated 50% of cases are HFpEF. Community screening programmes conducted in the UK broadly support this inference.^5,6^ The Heart Failure in Care Homes (HFinCH) study determined that 62.7% of those reviewed had HFpEF. The Echocardiographic Heart of England Screening Extension (Echoes-X) study adjudicated that 41.1% of those screened in general practice had HFpEF. Prevalence is therefore substantial and set to grow due to population aging and the burden of multi-morbidity. Recent analyses of heart failure clinic caseloads in England suggest that HFpEF is already the dominant form of heart failure presenting to clinical services (HFpEF vs. HFrEF: 33%/29% Sheffield, 46.6%/18.5% Salford).^7,8^

It is worth highlighting that these figures may mask the true prevalence of HFpEF, as significant biases and structural barriers that might prohibit presentation to a clinical setting have been documented.^9–11^ For example, work from the Optimising Management of Patients with Heart Failure with Preserved Ejection Fraction in Primary Care (OPTIMISE-HFpEF) programme suggests that knowledge and clinical suspicion levels are low.^9,10^ The *REF*er for *E*choca*R*diogram (REFER) study,^12^ which assessed the utility of natriuretic peptide thresholds used for diagnosing heart failure in the UK, determined that the comparatively high cut-points may result in one in five patients with heart failure not being referred, most of which were HFpEF. Lastly, a large pan-European survey^13^ has demonstrated that clinician confidence in diagnosing HFpEF is lower than for other forms of heart failure. In summary, there is a constellation of factors which might mean we are seeing ‘The Tip of the Iceberg’^14^ when it comes to HFpEF.

Within the UK, clinical services are commissioned by integrated care boards (ICBs) that are overseen by NHS England. Whilst services established under ICBs are responsible for delivering evidence-based care as dictated by the National Institute for Health and Care Excellence (NICE) guidance, the actual service provided will be agreed on a regional basis. This arrangement has resulted in significant variability, as evidenced by two studies which have shown that not all clinical services are commissioned to accept patients with HFpEF and that demand outstrips capacity.^15,16^ Other disparities in care are emerging; for example, recent research has shown that non-white patients with heart failure suffer from substantially higher rates of death and hospitalization than corresponding white patients.^17^

## Rationale

The growing volume of patients being diagnosed with HFpEF, coupled with recently identified effective treatments,^18^ calls for an exploration of any reported adaptions in UK heart failure clinical services. A contemporary review also highlighted discrepancies amongst HFpEF guidelines (e.g. different diagnostic echocardiographic thresholds and risk stratification algorithms).^19^ Consequently, there is scope for variability in service provision as practitioners draw upon other guidelines to supplement their knowledge and practice. To establish what services are offered to people with HFpEF in the UK, describe the format of those services, and assess how services compare to UK guideline recommendations, we conducted a scoping review.^20^ Whilst the review questions could also be addressed by a national audit or a survey, previous endeavours within clinical heart failure and cardiac rehabilitation services were acknowledged to be challenging due to the absence of a central register of services^15^ and low response rates.^21^ The results generated by this review are relevant internationally and capable of supporting the development of new clinical services to meet the complex needs of those with HFpEF.

## Objectives

This review paper will report on three aims: (i) to establish what services are currently offered to patients with HFpEF in the UK, (ii) to analyse and synthesize the format of HFpEF services, and (iii) to assess service provision against guideline recommendations. A fourth aim set out in the accompanying protocol publication^20^ is sequential (i.e. it builds on the results outlined here); therefore, it is not reported.

## Patient and public involvement

This review involved patient and public involvement and engagement (PPIE) input. Two PPIE contributors with HFpEF reviewed early reports and provided feedback on the clarity of the text and its concordance with their experiences.

## Methods

As per best practice, we have structured this report to comply with the Preferred Reporting Items for Systematic reviews and Meta-Analyses extension for Scoping Reviews (PRISMA-ScR) Checklist.^22^

### Protocol and registration

A protocol for this scoping review was published on the public Open Science Framework platform which can be accessed at https://osf.io/5gufq/. The scoping review protocol was also published in this journal.^20^

### Eligibility criteria

Justification for the eligibility criteria and the full Sample, Phenomenon of Interest, Design, Evaluation, Research type (SPIDER)^23^ criteria have been summarized in the protocol publication.^20^ In brief, any type of ‘literature’ was included if it met the SPIDER criteria outlined in *Table 1* and two further eligibility conditions: (i) available in the English language and (ii) published after 2013. An overview of the inclusion principles based on criteria is provided in *Table 1*.

**Table 1:** Abbreviated Sample, Phenomenon of Interest, Design, Evaluation, Research criteria

Criterion	Description
Sample	People with diagnosed HFpEF seen in any type of clinical service in the UK
Phenomenon of interest	Descriptions of supportive care/management provided to people with HFpEF
Design	Any publication that refers to ‘real-world’ HFpEF samples (e.g. non-experimental)
Evaluation	Multiple data fields covering demographic and disease management information
Research type	Published literature including posters, abstracts, audits, grey literature, hand-searched literature, and literature received following public appeal

### Information sources and selection of sources of evidence

The search strategy was three-pronged and included database searches, a search of Google Scholar, and data appeals hosted on social media and professional channels. Database searches were developed and managed in collaboration with an information specialist (IK). We separately searched six bibliographic databases [MEDLINE (via Ovid), Embase (via OVID), EMCARE (via Ovid), CINAHL (via EBSCO), Cochrane Library, Web of Science] from the period 1 January 2013 to 23 August 2023. Searches were updated on 3 December 2024. Search strategies were published as supplementary data with the review protocol.^20^

The first 500 results from Google Scholar were retrieved from a search that included the terms ‘HFpEF’, ‘Clinical Services’, and ‘UK’ and included the period 2013 until 3 December 2024. The same inclusion criteria were applied. Appeals were placed on social media (X and LinkedIn) and via professional network sites (British Society for Heart Failure), for heart failure specialists to share details of their current HFpEF clinical services (see Supplementary material online, *Figures S2* and *S3*). Twelve responses were received; however, only nine clinicians provided documentary evidence (clinical operations documents). Three clinicians provided short summaries within their response email, and these were deemed not robust enough to be included in the analysis.

### Selection of sources of evidence

The digital platform Rayyan^24^ was used to manage the screening process. All screening was performed blinded and in duplicate by six authors (F.F., M.G., M.E.H., S.T., A.D., R.C.A.). Title and abstract screening was undertaken to confirm possible eligibility. Any conflicts in screening adjudications were resolved by two authors (F.F. and R.C.A.). We retrieved the full texts of any provisionally eligible publications to confirm their eligibility against the SPIDER criteria.

### Data charting process

Data extraction was performed by one author (F.F.); 10% were checked for accuracy by a second author (R.C.A.). Reports that discussed the same sample, originating from the same heart failure service, were aggregated. Data were extracted only from unique reports; these could summarize the same services if they described a different sample (i.e. time point) or novel component/development of their clinical service. The data extraction template, which was designed to chart administrative and quantitative data, was built using Microsoft Excel software.^25^ The template was adapted following a small pilot (*n* = 5 articles). Reports were imported into NVivo software^26^ for extraction of narrative data presented in the Results of analysis and Discussion sections. These were analysed thematically by one author (F.F.); themes were verified by a second author (R.C.A.) via cross checking with the original publications.

### Data items

A broad range of data was considered including administrative, HFpEF characteristics, multi-morbidity, functional capacity, cognitive and frailty assessments, clinical service characteristics, and results/discussion data (see Supplementary material online, *Figure S1*). Whilst we obtained data on the items listed in the protocol, we do not report all of them due to extensive missing data. To assess concordance with the UK guidance, the current NICE heart failure guidelines (Chronic heart failure in adults: diagnosis and management NG106)^27^ were reviewed, and the recommendations for ‘Team working in the management of heart failure’ were tabulated. We considered it inappropriate to assess short reports against these criteria as their abbreviated nature would prohibit comprehensive reporting. However, as all full reports identified as observational, cohort, or reports of routinely collected data, it was deemed reasonable to expect reporting of these characteristics given the corresponding reporting guidelines recommend their inclusion.^28,29^

### Critical appraisal of individual sources of evidence

Critical appraisal is not mandated in scoping reviews; however, we assessed articles published in full against the Critical Appraisal Skills Programme (CASP) Checklist for Cohort Studies.^30^ Critical appraisal was performed to aid our interpretation of the trustworthiness of the data and as a means to comment on the quality of reports to guide future endeavours.

### Synthesis of results

Our synthesis was guided primarily by the systematic review without meta-analysis guidelines which determine seven synthesis reporting criteria that aid transparency.^31^ This was supplemented with guidance on narrative synthesis described by Popay *et al.*^32^

#### Grouping of studies

We grouped studies based on the type of report, i.e. peer-reviewed journal publication or descriptions of clinical operations. The rationale for these groupings was based on our assessment of the similarity and homogeneity of the report for synthesis.

#### Standardized metrics and transformation methods

We recorded data as reported; however, where necessary for combining or summarizing studies, we performed calculations (i.e. back transforming per cent to a number and vice versa) and substituted median for mean as per accepted practice.^33^

#### Synthesis methods

Numeric data were assessed via counts, ranges, per cent, and means. We synthesized data in relation to NICE recommendations for ‘Team working in the management of heart failure’.^27^ For assessment purposes, we grouped these NICE activities into the following three categories: staff, clinical activities, and referral; each category had multiple sub-categories (see *Table 2*). Articles were assessed against these criteria and marked as ‘yes’, ‘no’, or ‘unclear’. We did not extract specific details of what the clinic or service actually delivered in terms of care or how they achieved this. An example of decision-making is provided in Supplementary material online, *Box S1*.

**Table 2:** Concordance with National Institute for Health and Care Excellence recommendations

	Evidence to meet recommendation	
NICE recommendations	Articles: *n* (%)	Reports: *n* (%)
NICE category: staff		
Lead physician	9 (75%)	5 (56%)
Heart failure specialist nurse(s)	5 (42%)	9 (100%)
Specialist prescriber	9 (75%)	5 (56%)
NICE category: activities		
Diagnosis	12 (100%)	9 (100%)
Information giving (care plan)	11 (91%)	9 (100%)
Information giving (education)	6 (50%)	8 (89%)
Clinical review	12 (100%)	9 (100%)
Medication: optimization	11 (91%)	9 (100%)
Medication: monitoring and titration	11 (91%)	9 (100%)
Assessment and management: cardiac interventions	10 (83%)	5 (56%)
Assessment and management: acute decompensation	12 (100%)	9 (100%)
NICE category: referrals		
Cardiac rehabilitation	4 (33%)	8 (89%)
Older people’s services	1 (8%)	1 (11%)
Palliative care	13 (25%)	6 (67%)

All reports were imported into NVivo where textual descriptions (Results of analysis and Discussion sections) were analysed for conceptual overlap as per Popay *et al*.^32^ Similar data were grouped according to the latent construct; these concepts were labelled with descriptive codes and then organized into themes to synthesize learning.

#### Criteria used to prioritize results for summary and synthesis

We prioritized data that were more comprehensively reported, as we had more confidence in the validity of the findings. Conversely, we highlighted domains with significant under-reporting, as a means of identifying areas of weakness within this body of the literature.

#### Investigation of heterogeneity in reported effects

We visually inspected data for heterogeneity; there were insufficient quantities of data to undertake statistical assessment of heterogeneity.

#### Investigation of the certainty of evidence

We were unable to perform meta-analyses of effects; therefore, we were unable to generate statistics that would be amenable to interpretation by evidence certainty assessment frameworks.^34^

#### Description of the data presentation methods

We have described the two groups of data narratively, augmented by the thematic analysis.

## Results of analysis

**Figure 1:**
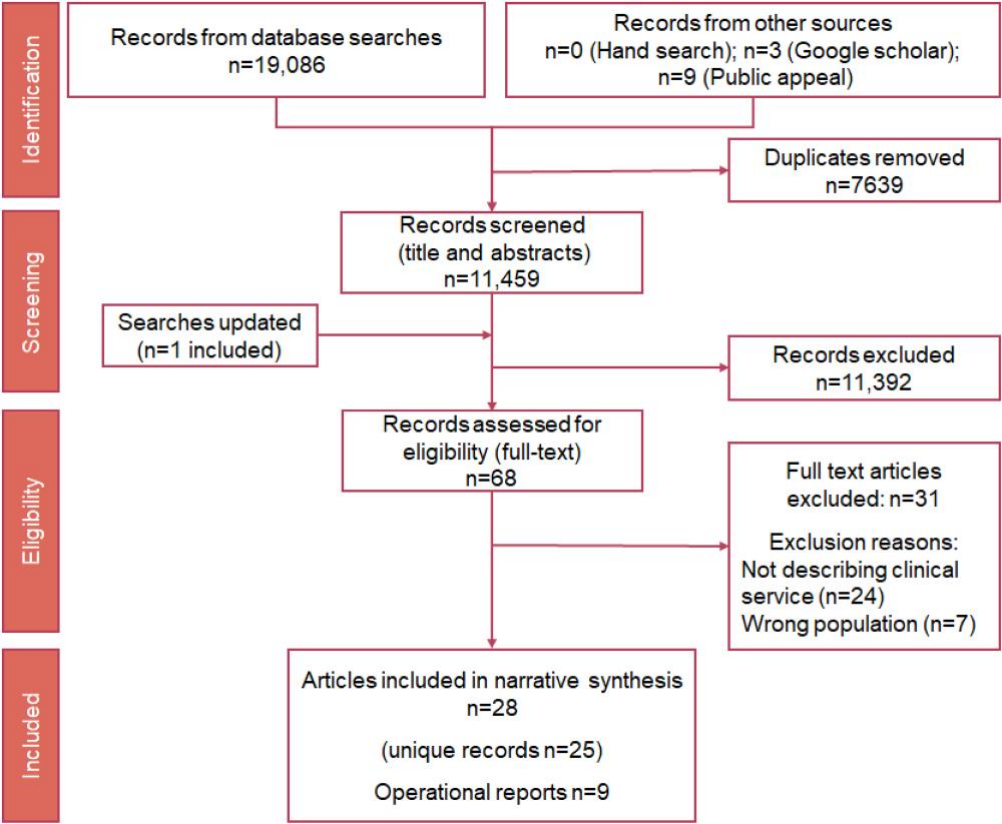
Preferred Reporting Items for Systematic reviews and Meta-Analyses flowchart.

We retrieved 11 459 potentially eligible reports. Updated searches retrieved one further article. We included 68 papers for full-text screening, and from these, 28 reports were included in the final analyses which represent 25 unique studies.^7,8,35–57^ We received nine operations documents via public appeal.^58–66^ A detailed overview is provided in the PRISMA flowchart (*Figure 1*). Tables with administrative, service, and clinical characteristics are provided in the Supplementary material (articles: Supplementary material online, *Tables S1*–*S3*; operations documents: Supplementary material online, *Tables S4*–*S6*).

### Results of bibliographic database and Google Scholar search

Data were extracted from 25 unique reports. These consisted of original research articles published in full (*n* = 12), abstracts (*n* = 12), and posters (*n* = 1) that ranged in publication date from 2013 to 2024. Most studies were conducted in England (20 out of 21); one report evaluated the introduction of a specialist heart failure pharmacist into a heart failure clinic in the UK. Across studies, 15 377 people were included with a mean age of 78.7 years, 35% male. Of these, 6789 (44%) were described as having diagnosed HFpEF.

Only six articles reported NTproBNP (mean 1380 pg/mL) and two reported BNP (mean 1329 ng/L). The majority documented the most common co-morbidities as individual disease counts and percentage; only one article^44^ reported using a validated measure of co-morbidity (Charlson Comorbidity Index^67^). The same article was the only one to describe undertaking an assessment of frailty.^44^ One article described performing cognitive assessments^36^; however, the measurement means were not documented. One article^44^ reported assessing frailty via Fried Criteria.^68^ Functional capacity assessments (via cardiopulmonary exercise test results, 6-min walk test distance, or other methods) were infrequently documented (unclear in *n* = 23, 92%). Clinical follow-up arrangements were poorly reported. Only four articles (16%) documented providing telephone follow-ups, 14 (56%) described providing ‘routine follow-up’, and a small proportion (*n* = 9, 36%) reported linking up with community outreach teams or providing community-based reviews. Overall descriptions of follow-up were sparse; therefore, we were unable to interrogate this data further.

Most services operated a ‘consultant-led’ clinic (*n* = 10, 42%), followed by ‘consultant oversight’ clinics (*n* = 5, 21%). One article defined their clinic type as ‘interdisciplinary’ (*n* = 1, 4%).^51^ In the remainder of the articles (*n* = 8, 33%), it was difficult to ascertain with any certainty clinic leadership. Six articles (25%) reported delivering multi-clinic types; for example, consultant-led clinics were often operated in conjunction with nurse-led clinics (*n* = 8). A proportion (*n* = 6, 24%) reported running a multidisciplinary service without specifying the composition of the multidisciplinary team. Details of the actual numbers of staff within each service were extracted, but due to infrequent reporting, we were unable to summarize these in a meaningful way.

A range of clinical and allied professionals were reported to be involved within heart failure clinics, in addition to heart failure specialist nurses and cardiologist/heart failure consultants. These included specialist heart failure pharmacists, geriatricians, nephrologists, palliative care specialists, anaemia nurse specialists, echocardiographers, cardiac physiologists, and pleural/ascitic care specialists. Two articles described having administrative and audit support (8.4%).

Twelve full articles were compared against NICE recommendations for ‘Team working in the management of heart failure’.^27^ Results of this assessment are presented in *Table 2*. Overall, most provided some evidence of complying with NICE guideline recommendations. Areas with the least concordance related to recommendations that the specialist heart failure multidisciplinary team should ‘directly involve, or refer people to, other services, including rehabilitation, services for older people and palliative care services, as needed’.

### Result of public appeal for operational information

Nine documents dated between 2017 and 2024 were included. Eight were assessed to be operational procedures documents and one described the development, operations, and outcomes of a pilot HFpEF clinical service. All described operating ‘nurse-led’ services; five stated these were accompanied by ‘consultant-led’ clinics. In the remaining reports (*n* = 4, 44%), service leadership was unclear. Most (*n* = 7, 78%) described operating multiple clinic types and delivering a multidisciplinary team service. In addition to a core multidisciplinary team, operational reports described collaborations with palliative care, frailty teams, psychological support services, mental health teams, community health teams, and specialist physiotherapists.

All operations reports described offering clinical assessment, treatment optimization, medication optimization, and routine follow-up. In contrast to published articles, there was greater documentation of patient education and rehabilitation referral (both 89%). Only one operational report described undertaking some form of functional assessments.

Operations documents were similarly compared with NICE recommendations for ‘Team working in the management of heart failure’ (*Table 2*). As with published articles, there was relatively good concordance with guidelines; however, it was often difficult to ascertain whether there was specialist clinician oversight of services. Analogous to the published articles, onward referral to rehabilitation, older people’s services, and palliative care appeared sub-optimal.

### Results of critical appraisal process

**Figure 2:**
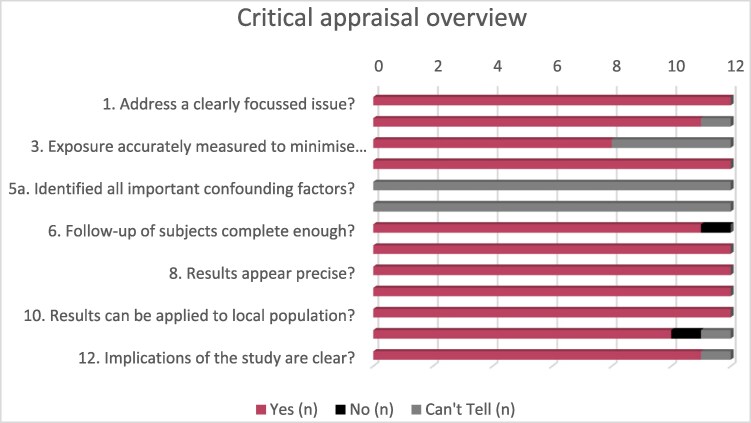
Critical appraisal overview.

Twelve full articles were assessed against the CASP Checklist for Cohort Studies (*Figure 2*).^30^ Articles complied with the majority of the appraisal standards for cohort studies; however, confounding factors were often not documented, nor were there descriptions of how these factors were accounted for in analysis (see Supplementary material online, *Table S7*).

### Results of thematic analysis

**Figure 3:**
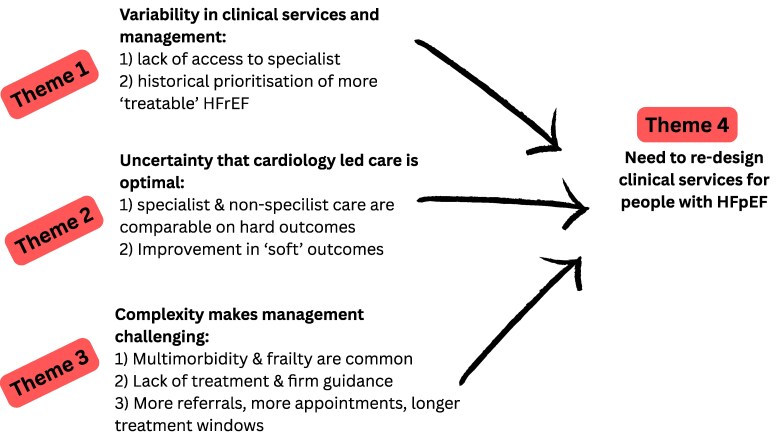
Thematic map.

Three themes and seven sub-themes were generated via thematic analysis that explained the overall findings and conclusions of the primary research (*Figure 3*): Theme 1, variability in services and management practices; Theme 2, uncertainty that cardiology-led care is optimal; and Theme 3, complexity makes management challenging. Collectively, these themes resulted in a widely endorsed concluding fourth theme, the ‘need for service re-design’. Quotes in support of these themes are presented; additional quotes are provided in the Supplementary material (see Supplementary material online, *Table S8*).

#### Theme 1: variability in services and management

Across articles, variability was reported in access and/or referral to specialist services. This could be the result of diagnostic thresholds, commissioning restrictions or other reasons not specified within the texts. Even if HFpEF services were commissioned, or people with HFpEF were being clinically assessed, there appeared to be a tendency to prioritize HFrEF patients for specialist care and rehabilitation.

*A substantial amount of patients with confirmed HFpEF were not accepted to the community heart failure specialist nurse because of insufficient evidence of signs and symptoms of heart failure. (Tavares et al. 2024)^42^*

*One quarter of HFpEF patients hospitalized were either not referred to cardiology or were discharged or died before receiving specialist input. (Cannata et al. 2023)^57^*

*In this analysis, 78.7% of patients with HFpEF received dedicated cardiology follow-up in the 12 months following their diagnosis. In contrast, a higher proportion of patients with HFrEF and HFmrEF received dedicated cardiology follow-up: 88.6% and 85.0%, respectively. (Migas et al. 2024)^8^*

#### Theme 2: uncertainty that cardiology-led care is optimal

Whilst one article indicated that specialist cardiology care for HFpEF reduced short-term mortality and readmission rates,^49^ larger and more recent articles have not.^45,57^ It is important to acknowledge that these articles included data that predated NICE approval of new therapies that have proven efficacy in reducing heart failure hospitalizations.^69^ Articles measuring alternative end points like initiation and up-titration of medications implied that heart failure services were effective in these domains. One publication questioned whether delivering care for HFpEF necessarily needs to be driven by cardiology specialists, such as heart failure specialist nurses.^47^

*In our analysis, long term survival in patients with HFpEF who received in-hospital specialist cardiology input was comparable to those who did not receive specialist input. (Cannata et al. 2023)^57^*

*Patients managed in the dedicated HFpEF clinic had a similar rate of cardiovascular and non-cardiovascular death and hospitalization compared with patients managed in the general clinic, despite greater input from the former clinic. (Tran et al. 2022)^45^*

*This observational study demonstrates that the HFSN-led community clinic has been effective in delivering evidence-based care for people with HFpEF. This included a substantial increase in initiation and/or up-titration of pharmacological therapies. (Tavares et al. 2024)^42^*

#### Theme 3: complexity makes management challenging

Three sub-themes contributed to this theme. Firstly, multi-morbidity and frailty were identified as common issues that drove complexity; secondly, the lack of treatment options and firm guidance on how to support patients with HFpEF were recognized as challenges to providing effective care. Lastly, complexity appeared to drive clinic activities and workload, as multiple articles suggested patients with HFpEF needed prolonged treatment, more or longer clinic appointments, and a high number of onward referrals. In one publication, this was linked to a reduction in care quality for people with HFrEF.^47^

*HFpEF patients were generally older, requiring higher diuretic doses and more prolonged treatment compared to other sub-groups. (Hassan et al. 2018)^54^*

*Patients with HFpEF often had their diuretics altered and, despite having fewer titrations than the HFrEF cohort, over 50% required more than six appointments. Investigation showed that these were not associated with medication titration and more likely to be associated with comorbidity management and social care. (Peplow and Rees 2024)^47^*

*[multiple] referrals were made to other specialties. The most common referral was to social services, followed by those classified as ‘other’, which included respiratory services, neurology, sleep studies, dietician services and care of the elderly. (Tavares et al. 2024)^42^*

#### Theme 4: need for service re-design

The latter three components generally resulted in articles concluding that service re-design alongside greater funding, training, and supportive infrastructures is needed. There appeared to be consensus that there was a need for a move away from cardiological focused care to a more multidisciplinary model that had greater integration of other specialities and social care.

*The current clinic model is not sufficiently structured to deliver the guideline-directed recommendations expected in the modern era of HFpEF. There is thus an imminent need to design a more comprehensive, patient centered HFpEF clinic that has the capacity to optimize the main cardiac and/or non-cardiac comorbidities. (Tran et al. 2022)^45^*

*The data also demonstrates an increased susceptibility of the HFpEF patient to non-CV emergency admission and points towards a need for a DMP to consider close clinical interaction with other medical specialists and indeed social and community services. (Murphy et al. 2017)^44^*

*Post discharge support pathways must be reviewed. Collaboration between specialist nurses and complex case management teams must be established to advance and evolve the discharge pathway. This should encourage multidisciplinary teams to work in a more integrated manner, with better multimorbidity management. (Pelow and Rees 2024)^47^*

## Discussion

This review has established that short reports outnumber full reports of HFpEF clinical services. The reasons behind this are unclear, but we speculate the following: (i) many clinicians may have focused on HFrEF services given that there is a longer history of research supporting interventions and consequently a larger pool of data for reporting and comparisons; (ii) surveys have demonstrated fewer services are commissioned to support HFpEF; therefore, there is less data available; and (iii) the type of data being described (retrospective reviews/audits) may have less appeal to journals. Clinical characteristics across articles were in-keeping with epidemiological reports with a high percentage of women (62%) and older adults (mean age 79 years) with elevated NTproBNP levels and high co-morbidity counts. We were unable to synthesize other outcomes of interest (frailty, functional capacity, NYHA classification, cognitive assessment) due to insufficient reporting.

Generally, the description of clinical services within the published literature was sparse, making it challenging to extract the composition and clinical activities with any certainty. There were fewer documentations of patient education, functional capacity assessment, and cardiac rehabilitation referral than expected. Whilst we have reported these data, we are unable to ascertain if this limited reporting is due to a need for article brevity or if these activities are excluded from these clinical services.

Descriptions of services were assessed against the broad guidance provided by NICE and marked as ‘yes, ‘no’, or ‘unclear’ in relation to whether they documented evidence that would meet the criteria in some way (see Supplementary material online, *Box S1*). On this basis, services do appear to be compliant with NICE recommendations; however, there is marked variability between descriptions, and we operated a low threshold for meeting a criterion. There is less clarity within published articles around arrangements for integration or referral to services for older people, palliative care, and rehabilitation. We did not abstract specific details of what and how the clinic actually delivered these components of care; therefore, we cannot comment on the quality of services offered. Had we used more detailed guidelines as the benchmark, for example, the European Society for Cardiology heart failure management guidelines,^70^ we may not have observed such high concordance.

Operational documents received through public appeals were all contributed by heart failure nurse specialists, which likely explains why the majority described nurse-led services. Within these descriptions, there was greater documentary evidence of providing patient education and referral to cardiac rehabilitation services. Concordance with NICE recommendations was on a par with the published articles. It is important to acknowledge that only services that are managing HFpEF would have contributed operational reports. Therefore, the data are inherently biased, and we cannot compare care to services that do not provide specialist support.

Documentation within published articles and operational reports of the range of additional specialties being co-opted to the HFpEF multidisciplinary team is particularly noteworthy. This evolution likely reflects the complexity of HFpEF patients who often require a holistic, multidisciplinary approach. However, it is also significant that few reports described links to elderly care (8% published articles, 11% operation reports), and only one-quarter of articles and one-third of operations reports detailed links to palliative care. Therefore, it would seem that there is still significant unmet need. Detailed descriptions of clinical services are therefore needed to generate insights into clinical presentation, care needs, and evaluation of outcomes.

Thematic analysis revealed important insights including the acknowledgement by current clinical service providers of variability in access to specialist cardiology services. Whilst most of these reports did not interrogate the reasons behind this, it might reflect the lack of capacity and preparedness within clinical services to provide tailored care for HFpEF patients. Qualitative research programmes would attest to the latter.^10,71,72^ The review focused on heart failure services that have reported or described the care they provide for people with HFpEF, and this may not reflect care across all services. Heart failure teams that have published on HFpEF services or shared operational documents may represent a standard not met by other services and the potential for a high level of unmet need.

Uncertainty surrounding the efficacy of clinical services to support those with HFpEF may also be a contributing factor in the variability observed. Two of the largest examinations thus far have not shown superiority of specialist heart failure care over non-specialist care,^57^ or specialist HFpEF care over standard heart failure clinical care.^45^ A broad spectrum of articles suggested that complexity, driven by frailty, multi-morbidity, and historic lack of treatments or specific guidance, made HFpEF complex and challenging to manage. This may explain why services have evolved to incorporate a broad range of augmentative specialist support as described here. In combination, the three themes variability, uncertainty, and complexity led to the conclusion that service re-design is needed to ensure services offer people with HFpEF holistic and multidisciplinary care.

Whilst not explicitly stated, it appears that HFpEF clinical services have been incorporated into existing HFrEF clinical care pathways, rather than being conceived from scratch. Consequently, it should not be a surprise that clinical service provision as reported does not match clinical need.^47^ Equally in heart failure guidelines, there remains a focus on disease-modifying pharmacological interventions which have evolved as a result of successful trials in HFrEF. Adding HFpEF care into this model without adequately adapting recommendations to the needs of people with HFpEF seems sub-optimal. One article that has adapted guidelines to incorporate a HFpEF attuned mnemonic (A, anaemia/atrial fibrillation/sleep apnoea; B, blood pressure control, body mass index; C, chronic kidney disease and chronic obstructive pulmonary disease; D, diabetes control; E, exercise rehabilitation; F, frailty assessment and need for advanced care planning) has shown a trend towards lower 3-month all-cause readmissions.^39^

## Conclusion

Despite the large and increasing number of patients presenting with HFpEF, there are very few publications that describe real-world practice at the clinical service level. Of those that have described their services via publication or within operational reports shared with us, it would appear that care is broadly compliant with NICE recommendations. However, we have identified that care is variable, not necessarily tailored to deliver better long-term outcomes in HFpEF, and difficult to implement in practice. As a result, clinical teams who have examined their services have suggested that there is a need to re-think and re-design care pathways. New pathways might be less cardio-centric and integrate a wide range of specialism and social care. Clinical services are already evolving, as evidenced by our assessment of the range of specialities/specialists HFpEF services have embedded within their care teams. It is important that national clinical guidance evolves in relation to the findings presented here, which reflect real-world clinical practice. To build truly bespoke services, there is a need to avoid forcing HFpEF services into HFrEF pathways. Instead, detailed programmes of research, which might aptly be conducted by heart failure specialist nurses, are required to help develop tailored and effective services for this growing patient population.

## Supplementary Material

zvaf143_Supplementary_Data

## Data Availability

Data will be made available upon reasonable request.

## References

[1] Conrad N, Judge A, Tran J, Mohseni H, Hedgecott D, Crespillo AP, et al Temporal trends and patterns in heart failure incidence: a population-based study of 4 million individuals. The Lancet 2018;391:572–580. 10.1016/S0140-6736(17)32520-5 PMC5814791 29174292

[2] Campbell P, Rutten FH, Lee MM, Hawkins NM, Petrie MC. Heart failure with preserved ejection fraction: everything the clinician needs to know. Lancet 2024;403:1083–1092. 38367642 10.1016/S0140-6736(23)02756-3

[3] Savarese G, Becher PM, Lund LH, Seferovic P, Rosano GMC, Coats AJS. Global burden of heart failure: a comprehensive and updated review of epidemiology. Cardiovasc Res 2023;118:3272–3287. 35150240 10.1093/cvr/cvac013

[4] Dunlay SM, Roger VL, Redfield MM. Epidemiology of heart failure with preserved ejection fraction. Nat Rev Cardiol 2017;14:591–602. 28492288 10.1038/nrcardio.2017.65

[5] Hancock HC, Close H, Mason JM, Murphy JJ, Fuat A, Singh R, et al High prevalence of undetected heart failure in long-term care residents: findings from the Heart Failure in Care Homes (HFinCH) study. Eur J Heart Fail 2013;15:158–165. 23112002 10.1093/eurjhf/hfs165 PMC3547366

[6] Davies M, Hobbs F, Davis R, Kenkre J, Roalfe AK, Hare R, et al Prevalence of left-ventricular systolic dysfunction and heart failure in the Echocardiographic Heart of England Screening study: a population based study. Lancet 2001;358:439–444. 11513906 10.1016/s0140-6736(01)05620-3

[7] Garg P, Dakshi A, Assadi H, Swift AJ, Naveed U, Fent G, et al Characterisation of the patients with suspected heart failure: experience from the SHEAF registry. Open Heart 2021;8:e001448. 33431617 10.1136/openhrt-2020-001448 PMC7802648

[8] Migas S, Ellis ML, Wrona B, Rivero Sanz E, Brownrigg J, Strauss O, et al Missed opportunities in heart failure diagnosis and management: study of an urban UK population. ESC Heart Fail 2024;11:2200–2213. 38627992 10.1002/ehf2.14766 PMC11287321

[9] Hossain MZ, Chew-Graham CA, Sowden E, Blakeman T, Wellwood I, Tierney S, et al Challenges in the management of people with heart failure with preserved ejection fraction (HFpEF) in primary care: a qualitative study of general practitioner perspectives. Chronic Illn 2021;18:1–16. 10.1177/1742395320983871 PMC9163769 33401942

[10] Sowden E, Hossain M, Chew-Graham C, Blakeman T, Tierney S, Wellwood I, et al Understanding the management of heart failure with preserved ejection fraction: a qualitative multiperspective study. Br J Gen Pract 2020;70:e880–e889. 33139334 10.3399/bjgp20X713477 PMC7643822

[11] Brooman-White R, Blakeman T, McNab D, Deaton C. Informing understanding of coordination of care for patients with heart failure with preserved ejection fraction: a secondary qualitative analysis. BMJ Qual Saf 2023;33:232–245. 10.1136/bmjqs-2023-016583 37802647

[12] Taylor CJ, Roalfe AK, Iles R, Hobbs FR, investigators R, Barton P, et al Primary care REFerral for EchocaRdiogram (REFER) in heart failure: a diagnostic accuracy study. Br J Gen Pract 2017;67:e94–e102. 27919937 10.3399/bjgp16X688393 PMC5308123

[13] Howlett J, Comin-Colet J, Dickstein K, Fuat A, Polzl G, Delaney S. Clinical practices and attitudes regarding the diagnosis and management of heart failure: findings from the CORE needs assessment survey. ESC Heart Fail 2018;5:172–183. 28921886 10.1002/ehf2.12205 PMC5793971

[14] Deaton C, Edwards D, Malyon A, Zaman MJ. The tip of the iceberg: finding patients with heart failure with preserved ejection fraction in primary care. An observational study. BJGP Open 2018;2:bjgpopen18X101606. 10.3399/bjgpopen18X101606 PMC6189778 30564739

[15] Graves B, Hartshorne-Evans N. Heart failure specialist nurse care: more questions than answers!. Br J Cardiol 2019;26:86–87.

[16] Kwok CS, Piper SE, Deaton C, Masters J, Duckett S. Heart failure services from the hospital perspective in the UK: a cross-sectional survey. Br J Cardiol 2025;32:14–18. 10.5837/bjc.2025.001 PMC13343823 42422309

[17] Fox S, Champsi A, Hardy E, Bunting K, Kotecha D. The impact of ethnicity on clinical outcomes in patients with heart failure and atrial fibrillation. Eur Heart J 2024;45:ehae666.885.

[18] Vaduganathan M, Docherty KF, Claggett BL, Jhund PS, de Boer RA, Hernandez AF, et al SGLT-2 inhibitors in patients with heart failure: a comprehensive meta-analysis of five randomised controlled trials. Lancet 2022;400:757–767. 36041474 10.1016/S0140-6736(22)01429-5

[19] Mahmood A, Dhall E, Primus CP, Gallagher A, Zakeri R, Mohammed SF, et al Heart failure with preserved ejection fraction management: a systematic review of clinical practice guidelines and recommendations. Eur Heart J—Qual Care Clin Outcomes 2024;10:571–589. 38918060 10.1093/ehjqcco/qcae053 PMC11537231

[20] Forsyth F, Deaton C, Kalra PR, Green M, Harrison ME, Tavares S, et al What services are currently provided to people with HFpEF in the UK and what are their components? A protocol for a scoping literature review. Eur J Cardiovasc Nurs 2024;24:83–88. 10.1093/eurjcn/zvae119 PMC11781374 39186550

[21] Dalal HM, Wingham J, Palmer J, Taylor R, Petre C, Lewin R, et al Why do so few patients with heart failure participate in cardiac rehabilitation? A cross-sectional survey from England, Wales and Northern Ireland. BMJ Open 2012;2:e000787. 10.1136/bmjopen-2011-000787 PMC3323807 22454188

[22] Tricco AC, Lillie E, Zarin W, O'Brien KK, Colquhoun H, Levac D, et al PRISMA extension for scoping reviews (PRISMA-ScR): checklist and explanation. Ann Intern Med 2018;169:467–473. 30178033 10.7326/M18-0850

[23] Methley AM, Campbell S, Chew-Graham C, McNally R, Cheraghi-Sohi S. PICO, PICOS and SPIDER: a comparison study of specificity and sensitivity in three search tools for qualitative systematic reviews. BMC Health Serv Res 2014;14:579. 25413154 10.1186/s12913-014-0579-0 PMC4310146

[24] Ouzzani M, Hammady H, Fedorowicz Z, Elmagarmid A. Rayyan-a web and mobile app for systematic reviews. Syst Rev 2016;5:210. 27919275 10.1186/s13643-016-0384-4 PMC5139140

[25] Microsoft Corporation. Microsoft Excel [Internet]. 2018. Available from: https://office.microsoft.com/excel.

[26] QSR International Pty Ltd. NVivo (Version 12). 2018.

[27] National Institute for Health and Care Excellence . Chronic heart failure in adults: diagnosis and management (NG108). 2018. Available from: https://www.nice.org.uk/guidance/ng106. 30645061

[28] von Elm E, Altman DG, Egger M, Pocock SJ, Gotzsche PC, Vandenbroucke JP, et al The Strengthening the Reporting of Observational Studies in Epidemiology (STROBE) statement: guidelines for reporting observational studies. J Clin Epidemiol 2008;61:344–349. 18313558 10.1016/j.jclinepi.2007.11.008

[29] Benchimol EI, Smeeth L, Guttmann A, Harron K, Moher D, Petersen I, et al The REporting of studies Conducted using Observational Routinely-collected health Data (RECORD) statement. PLoS Med 2015;12:e1001885. 26440803 10.1371/journal.pmed.1001885 PMC4595218

[30] CASP Programme . CASP Checklist for: Cohort Studies [online]. 2024. Available from: https://casp-uk.net/casp-tools-checklists/ (accessed 10/12/2024).

[31] Campbell M, McKenzie JE, Sowden A, Katikireddi SV, Brennan SE, Ellis S, et al Synthesis without meta-analysis (SWiM) in systematic reviews: reporting guideline. BMJ 2020;368:l6890. 31948937 10.1136/bmj.l6890 PMC7190266

[32] Popay J, Roberts H, Sowden A, Petticrew M, Arai L, Rodgers M, et al Guidance on the Conduct of Narrative Synthesis in Systematic Reviews A Product from the ESRC Methods Programme. 2006. 10.13140/2.1.1018.4643

[33] Higgins JPT, Thomas J, Chandler J, Cumpston M, Li T, Page MJ, et al Cochrane Handbook for Systematic Reviews of Interventions version 6.4 (updated August 2023). Cochrane, 2023. 2022. Available from: www.training.cochrane.org/handbook (accessed 24/05/2024).

[34] Guyatt GH, Oxman AD, Vist GE, Kunz R, Falck-Ytter Y, Alonso-Coello P, et al GRADE: an emerging consensus on rating quality of evidence and strength of recommendations. Bmj 2008;336:924–926. 18436948 10.1136/bmj.39489.470347.AD PMC2335261

[35] Thompson A, Crilley J, Wilson D, Hungin A, Fuat A, Murphy J. 24 an epidemic of HFPEF? Heart 2016;102:A15–A16.

[36] Simms J, Irani T, Schiff R. 38 HEART FAILURE IN THE OLDER PERSON: IS THERE STILL A PLACE FOR THE GERIATRICIAN? Age Ageing 2014;43:i9–i9.

[37] Sinclair H, Ackrill M, Holdsworth H, Chase C, Guillen M, Bowman L, et al 88 rapid access heart failure clinic: impact of a physiologist-delivered service in a UK district general hospital. Heart 2019;105:A74–A74.

[38] Shah J, Paz E, Elias E, Alimo A, Sharma S, Elizondo I, et al 114 the challenges and opportunities of starting a heart failure virtual ward, experience from London North West University Healthcare NHS Trust. Heart 2022;108:A85–A85.

[39] Murphy N, Duvva D, Kelly A-M, Nyjo S, Bower C, Jackson C, et al 168 can the use of a structured management approach using abcdef mnemonic to manage multi-morbidity in HFpEF improve clinical outcomes? Heart 2024;110:178–178.37714697

[40] Bolam H, Kalra P, Guha K, Morton D. Abstract: P2005—the impact of a clinical educational and self-care intervention in patients with heart failure with preserved ejection fraction. Eur J Heart Fail 2019.

[41] Guha K, Allen CJ, Chawla S, Pryse-Hawkins H, Fallon L, Chambers V, et al Audit of a tertiary heart failure outpatient service to assess compliance with NICE guidelines. Clin Med (Lond) 2016;16:407–411. 27697799 10.7861/clinmedicine.16-5-407 PMC6297303

[42] Tavares S, Kanaganayagam G, Lampridou S, Phuyal U, Singh H, Forsyth F. Characteristics and outcomes of patients with heart failure with preserved ejection fraction referred to a community specialist nurse-led clinic. Br J Card Nurs 2024;19:1–14.

[43] Zheng A, Cowan E, Mach L, Adam RD, Guha K, Cowburn PJ, et al Characteristics and outcomes of patients with suspected heart failure referred in line with National Institute for Health and Care Excellence guidance. Heart 2020;106:1579–1585. 32690621 10.1136/heartjnl-2019-316511 PMC7525790

[44] Murphy TM, Waterhouse DF, James S, Casey C, Fitzgerald E, O'Connell E, et al A comparison of HFrEF vs HFpEF’s clinical workload and cost in the first year following hospitalization and enrollment in a disease management program. Int J Cardiol 2017;232:330–335. 28087180 10.1016/j.ijcard.2016.12.057

[45] Tran P, Long T, Smith J, Kuehl M, Mahdy T, Banerjee P. Developing a contemporary community clinic for patients with heart failure with preserved ejection fraction within the current National Health Service model. Open Heart 2022;9:e002101. 36332941 10.1136/openhrt-2022-002101 PMC9639156

[46] Morton G, Philip L, Gilpin T, Chan PE, Guha K, Kalra PR. Does specialist review for patients with suspected heart failure predict better outcomes? An observational study on the utility of compliance with NICE guidelines. BMJ Open 2018;8:e021856. 10.1136/bmjopen-2018-021856 PMC6112396 30139902

[47] Peplow J, Rees S. An evaluation of a service expansion to include patients with heart failure with preserved ejection fraction. Br J Card Nurs 2024;19:1–10.

[48] Griffiths A, Taylor J, McRae D. Evaluation of a specialist pharmacist led diagnostic chronic heart failure clinic, In. European Journal of Heart Failure. NJ USA: WILEY; 2020. p258–258.

[49] Hawley A, He J, Crabtree A, Iacovides S, Keeling P. The impact of an integrated heart failure service in a medium-sized district general hospital. Open Heart 2020;7:e001218. 32393657 10.1136/openhrt-2019-001218 PMC7223459

[50] Al-Mohammad A, Watt V, O'Toole L, Hall I, Yates L. Insights into the epidemiology of incident heart failure (HF): outcomes of rapid HF access clinic applying the NICE guidelines. Eur Heart J 2013;34:P4225.

[51] Nguyen M, Rumjaun S, Lowe-Jones R, Ster IC, Rosano G, Anderson L, et al Management and outcomes of heart failure patients with CKD: experience from an inter-disciplinary clinic. ESC Heart Fail 2020;7:3225–3230. 32652822 10.1002/ehf2.12796 PMC7524254

[52] Horan C, Bower C, Kennedy J, O'Pray A, Crilly M, Thronton-Clay H, et al Outcomes of heart failure specialist nurse delivered consultant led community virtual heart failure multidisciplinary team meetings during the first peak of the COVID-19 pandemic. 2021. https://esc365.escardio.org/Presentation/233352/abstract (accessed 01/10/2024).

[53] Monteiro C, Cojoianu A, Savage R, Bone R, Hammond C, Gamble J, et al P214 clinical audit of in-patient echocardiography in acute heart failure: real world data from a tertiary hospital. Eur Heart J—Cardiovasc Imaging 2020;21.

[54] Hassan S, Lee C, Beleznai T, Nyjo S, Jackson C, Fenlon K, et al P276 heart failure specialist nurse-led day case ambulatory management with intravenous diuretics reduces hospitalisations for acute decompensated heart failure irrespective of ejection fraction. Eur Heart J 2018;39:ehy564.P276.

[55] Doleman F, Santon M, Clewes J, Laithwaite C, McIntosh R, Ahmed H, et al P2593Evaluation of an ambulatory heart failure service—a 2 years’ experience. Eur Heart J 2019;40.

[56] Dulai R, Sheikh AS, Qureshi A, Katechia S, Peysakhova Y, Johns M, et al Prevalence, clinical characteristics and outcomes of HF with preserved versus reduced ejection fraction. Br J Cardiol 2016;23:1–40.

[57] Cannata A, Badawy L, Anyu AT, Samways J, Sweeney M, Jordan-Rios A, et al The prognostic impact of specialist cardiology input in patients admitted for heart failure and normal ejection fraction. ESC Heart Fail 2023;10:2648–2655. 37357540 10.1002/ehf2.14440 PMC10375143

[58] Kings Health Partners . Heart Failure Specialist Nurse Service Operational Procedures Acute and Community Setting. 2017.

[59] Imperial College Healthcare . Chronic Heart Failure Guide.

[60] Sussex Health and Care Partners . Heart Failure Diagnostic and Treatment Pathway in Primary Care. 2021.

[61] Oxford University Hospitals . Heart Failure Guidance Summary for GPs. 2021.

[62] University Hospitals of North Midlands . Standard Operating Procedure for the Ambulatory Heart Failure Units. 2020.

[63] Ayrshire and Arran NHS Trust . Developing a pathway for individuals with HFpEF - Project Evaluation. 2024.

[64] Wiltshire Health and Care . Standard Operational Procedure - Community Heart Failure Service Ops. 2024.

[65] Leicestershire Partnership . Standard operating procedure - Heart Failure Specialist Nursing Service Leicestershire Partnership NHS Trust. 2024.

[66] West Hertfordshire NHS Trust . West Hertforshire proposed model for heart failure nurse service West Herts. 2019.

[67] Charlson ME, Pompei P, Ales KL, MacKenzie CR. A new method of classifying prognostic comorbidity in longitudinal studies: development and validation. J Chronic Dis 1987;40:373–383. 3558716 10.1016/0021-9681(87)90171-8

[68] Fried LP, Tangen CM, Walston J, Newman AB, Hirsch C, Gottdiener J, et al Frailty in older adults: evidence for a phenotype. J Gerontol—Ser A Biol Sci Med Sci 2001;56:M146–M156. 10.1093/gerona/56.3.m146 11253156

[69] National Institute for Health and Care Excellence. Dapagliflozin for treating chronic heart failure with preserved or mildly reduced ejection fraction (TA902). 2023. 40146869

[70] McDonagh TA, Metra M, Adamo M, Gardner RS, Baumbach A, Bohm M, et al 2021 ESC guidelines for the diagnosis and treatment of acute and chronic heart failure. Eur Heart J 2021;42:3599–3726. 34922348 10.1093/eurheartj/ehab853

[71] Pearson CR, Forsyth F, Khair E, Sowden E, Borja Boluda S, Deaton C, et al Keeping the plates spinning': a qualitative study of the complexity, barriers, and facilitators to caregiving in heart failure with preserved ejection fraction. Eur J Cardiovasc Nurs 2023;22:141–148. 35714068 10.1093/eurjcn/zvac027

[72] Forsyth F, Blakeman T, Burt J, Chew-Graham CA, Hossain M, Mant J, et al Cumulative complexity: a qualitative analysis of patients’ experiences of living with heart failure with preserved ejection fraction. Eur J Cardiovasc Nurs 2022;22:529–536. 10.1093/eurjcn/zvac081 36073202

