# Supplementary material for: What services are currently provided to people with heart failure with preserved ejection fraction in the UK and what are their components? A systematic scoping review
Source: Eur J Cardiovasc Nurs. 2025 Jul 17;25(2):227–36. doi: 10.1093/eurjcn/zvaf143 (PMC13126105; doi:10.1093/eurjcn/zvaf143)
Supplement: zvaf143_Supplementary_Data [file zvaf143_supplementary_data.docx]

**Table of Contents**

[Figure 1: Lay summary of findings shared with patient and public involvement group members 2](#_Toc196475372)

[Figure 2: Appeal for data on X (formally known as Twitter) 3](#_Toc196475373)

[Figure 3: Appeal for data on British Society for Heart Failure 4](#_Toc196475374)

[Box 1:Example of grading against NICE categories 5](#_Toc196475375)

[Table 1: Clinical service description 38](#_Toc196475376)

[Table 2: Clinical service / management characteristics 48](#_Toc196475377)

[Table 3: Sample characteristics 51](#_Toc196475378)

[Table 4: Operations documents description 54](#_Toc196475379)

[Table 5: Operations documents clinical service / management 1 55](#_Toc196475380)

[Table 6: Operations documents clinical service / management 2 56](#_Toc196475381)

[Table 7 Critical Appraisal Assessment 57](#_Toc196475382)

[Table 8: Additional quotes in support of themes 59](#_Toc196475383)

[References 60](#_Toc196475384)

## Figure 1: Lay summary of findings shared with patient and public involvement group members


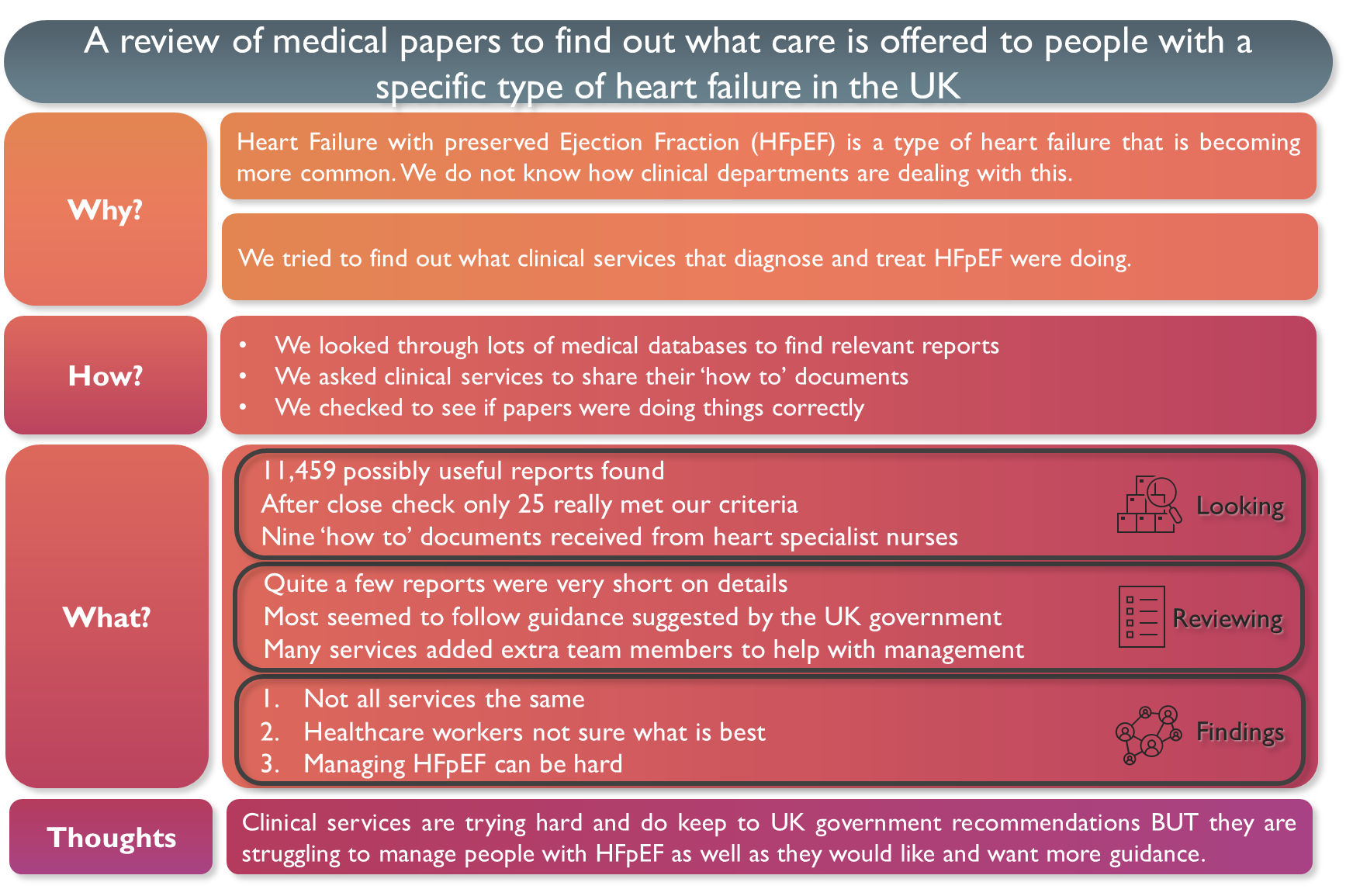


## Figure 2: Appeal for data on X (formally known as Twitter)


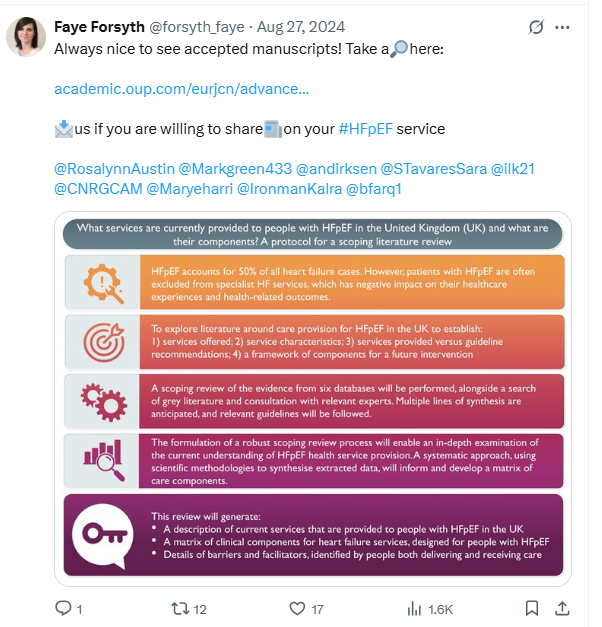


## Figure 3: Appeal for data on British Society for Heart Failure

| **Description of clinical service from Tran et al.**  University Hospitals Coventry and Warwickshire (UHCW) NHS Trust was one of the first UK centres to establish a dedicated HFpEF community clinic, which was set up before the advent of novel cardiometabolic therapies. Instead, the main pillars of HFpEF management consisted of education, exercise, volume control, management of blood pressure and atrial fibrillation. It is important to appreciate that when this clinic was conceived, there was neither a structured, funded provision of care, nor HF specialist nurse (HFSN) support, unlike the HFrEF service.  UHCW is a tertiary cardiac centre that runs daily consultant-led general cardiology clinics, where a mixture of patients with HFrEF and HFpEF are seen by both HF and non-HF specialists including trainee registrars. Prior to the global viral pandemic when all consultations were held face to face, a specific HFpEF community clinic was set up, which was solely led by a consultant cardiologist specialising in HF in the City of Coventry Health  Centre, UK. The dedicated HFpEF clinic was a smaller clinic which saw approximately up to 6 suspected HFpEF patients every week as opposed to the general clinic which was a larger clinic of around 24 patients seeing a mixture of HF patients of all types including HFpEF. As the latter clinic was busier, not all patients seen by cardiology registrars in that clinic were necessarily reviewed or discussed with the consultant cardiologist.  The service evaluation focused on interventions delivered to patients during their first consultation. In both clinics, it was clear that most interventions were cardiology-focused, namely congestion management (64% patients), review of antihypertensives (86%) and rate or rhythm control of AF with either cardioversion or referral for ablation (48%). Non-pharmacological measures consisted of education on fluid (to ~1.5 L/day) and salt restriction (<2400 mg/day), advice on moderate-intensity exercise according to national guidelines, including referrals to cardiac rehabilitation for personalized exercise programmes. Patients seen in the dedicated HFpEF service were significantly more likely to receive these interventions than those attending the general clinic. | |
| --- | --- |
| NICE Category: Staff | |
| Lead Physician | Yes |
| Heart Failure Specialist Nurse(s) | No |
| Specialist Prescriber | Yes |
| NICE Category: Activities | |
| Diagnosis | Yes |
| Information giving (care plan) | Yes |
| Information giving (education) | Yes |
| Clinical review | Yes |
| Medication: optimisation | Yes |
| Medication: monitoring and titration | Yes |
| Assessment & Management: cardiac interventions | Yes |
| Assessment & Management: acute decomposition | Unclear |
| NICE category: referrals | |
| Cardiac rehabilitation | Yes |
| Older people’s services | Unclear |
| Palliative care | Unclear |

## Box 1:Example of grading against NICE categories

| **Authors** | **Title** | **Year** | **Journal** | **Publication Type** | **Sub-type** | **Aim** | **Clinic Location** | **Descrition of Clinic Types 1** | **Descrition of Clinic Types 2** | **Multiple clinic types** | **MDT input / approach** | **HF OR Cardiology Consultant** | **Consultants (n)** | **HFSN (n)** | **Medical trainees** | **Pharmacist** | **Other** | **Service links** | **Service links** | **Service links** | **Service described 1** | **Service described 2** | **Service described 3** | **clinical assessment** | **cardiological optimisation** | **medication optimisation** | **patient education** | **functional assessment** | **rehab referral** | **telephone triage** | **community outreach / review** | **routine follow-up** | **Sample (n)** | **Age (all, years)** | **Sex (male)** | **HFpEF (n)** | **HFpEF (%)** |
| --- | --- | --- | --- | --- | --- | --- | --- | --- | --- | --- | --- | --- | --- | --- | --- | --- | --- | --- | --- | --- | --- | --- | --- | --- | --- | --- | --- | --- | --- | --- | --- | --- | --- | --- | --- | --- | --- |
| Guha, K. and Allen, C. J. and Chawla, S. and Pryse-Hawkins, H. and Fallon, L. and Chambers, V. and Vazir, A. and Lyon, A. R. and Cowie, M. R. and Sharma, R. | Audit of a tertiary heart failure outpatient service to assess compliance with NICE guidelines | 2016 | Clinical Medicine | full paper | audit | To compare 2013 audit data to 2011 audit data on admissions to a tertiary cardiac centre. | London | Consultant led | Nurse led | yes | yes | Y | 4 | 3 | multiple | yes | ns | Cardiac imaging | Palliative care | ns | Patient monitoring, including assessment of clinical (functional capacity, fluid status, heart rhythm etc) and biochemical (serum urea, creatinine, electrolytes and estimated glomerular filtration rate (eGFR)) parameters | Prescription of evidence-based medication in HFrEF patients at maximum tolerated doses, specifically a beta-blocker and ACEi/ARB licensed for HF alongside and MRA if indicated. | Rehabilitation provision, including the education of all patients regarding the benefits of exercise in chronic HF and referral to an evidence-based rehabilitation programme. | Y | Y | Y | Y | Y | Y | UC | UC | Y | 500 | 68 | 352 | 95 | 19 |
| Murphy, T. and Waterhouse, D. and James, S. and Casey, C. and Fitzgerald, E. and O'Connell, E. and Watson, C. and Gallagher, J. and Ledwidge, M. and McDonald, K. | A Comparison of Hfref Vs Hfpef's Clinical Workload and Cost in the First Year Following Hospitalisation and Enrollment in a Disease Management Program | 2017 | International Journal of Cardiology | full paper | Retrospective observational | To investigate resource utilization and cost comparing heart failure with reduced ejection fraction and HFpEF. | Dublin | Consultant led | Nurse led | yes | yes | UC | ns | ns | ns | ns | ns | ns | ns | ns | Management includes complete work up of the cause of HF, assessment of the likely precipitant of admission and initiation of appropriate therapy. Once clinically improved, comprehensive one-on-one education with the patient is initiated regarding standard self-care issues. Contact is also made with the next of kin for education on what to expect on discharge and howto recognise features of decompensation. Discharge planning is initiated when the patient is deemed euvolaemic, or as near to this state as can be effectively achieved. | The outpatient programme is run from a chronic ambulatory care facility, which provides scheduled and unscheduled clinic access Monday to Friday 9 am–5 pmand unscheduled access at weekends through the on-call cardiology service in the hospital. All patients have three scheduled assessment visits during the 3-month period post discharge; within two weeks of discharge (exact timing determined by clinical stability on discharge), and again at 6 weeks and 12 weeks. Nurse-initiated phone consultations (outbound calls) are made to the patients within 48-hours of hospital discharge and then weekly for 12 weeks except on clinic visit weeks. The purpose of these calls is to assess well-being and address any patient concerns. Additionally, these outbound calls provide an opportunity for our HF nurse specialists to engage with the patient on matters such as medication adherence, self-care, body weight measurement, exercise and adherence to any dietary restrictions. | Patients, their carers or general practitioners can contact the unit outside of these scheduled contacts for assistance in management of features suggestive of emerging clinical deterioration. These calls are triaged by a nurse specialist and a decision ismade tomanage remotely or review the patient at the clinic that day if indicated (unscheduled clinic visit). | Y | Y | Y | Y | UC | UC | Y | UC | Y | 1292 | 74.5 | 759 | 413 | 32 |
| Hassan, S. and Lee, C. and Beleznai, T. and Nyjo, S. and Jackson, C. and Fenlon, K. and Llewellyn, J. and Douglas, H. and Kanagala, P. and Sankaranarayanan, R. | Heart failure specialist nurse-led day case ambulatory management with intravenous diuretics reduces hospitalisations for acute decompensated heart failure irrespective of ejection fraction | 2018 | European Heart Journal | abstract | Retrospective observational | To describe the development of a nurse led ambulatory HF unit | Liverpool | Consultant oversight | Nurse led | yes | yes | UC | ns | ns | ns | ns | ns | Renal | Pleural / ascities specialist | Palliative care | Our ambulatory HF unit (AHFU) receives referrals from emergency department, primary care and community teams. The unit provides intravenous (IV) bolus furosemide (if required) at 4mg/mt in an ambulatory setting. A multidisciplinary approach is used through regular input from day-case renal, pleural and ascites clinics, as well as palliative care team. We analysed the efficacy of ambulatory management of ADHF and compared the outcomes based on the ejection fraction (EF). | na | na | Y | Y | UC | UC | UC | UC | UC | UC | UC | 335 | 80 | ns | 117 | 35 |
| Hawley, A. and He, J. and Crabtree, A. and Iacovides, S. and Keeling, P. | The impact of an integrated heart failure service in a medium-sized district general hospital | 2020 | Open Heart | full paper | Retrospective observational | To assess the impact of a new integrated heart failure service (IHFS) in a medium-sized district general hospital (DGH) on heart failure (HF) mortality, readmission rates, and provision of HF care. | Torbay & South Devon | Consultant led | Nurse led | yes | yes | Y | 2 | 5 | ns | ns | administrative & audit support | Renal | Palliative care | elderly care | The IHFS takes a multidisciplinary approach and comprises two cardiologists with an interest in HF, five specialist heart failure nurses (HFNs) (two were already employed within primary care and three (2.4 whole-time-equivalent (WTE)) were new appointments) together with administrative and audit support. HFN training involved an 18-month rotation between the hospital and community prior to establishment of a single hospital-based, and four community-based HFNs (total 4.4 WTE). | The IHFS actively identifies patients admitted with uspected HF using B-type natriuretic peptide (BNP) and automated email alerts for patients with known HF. The HFNs review these patients and discuss them with the ‘cardiologist of the week’. Together, they identify patients suitable for transfer to the cardiology ward. Furthermore, all in-patients are discussed formally at the weekly HF multidisciplinary team (MDT). This provides all patients with specialist consultant input, either by face-to-face review on the HF ward, or in discussion with the HFNs. | Following discharge (pre-IHFS and post-IHFS), patients are scheduled for urgent outpatient consultant clinic review, which usually occurs within 6 weeks (due to high demand). However, the new IHFS aims to also provide specialist HFN review in the community for all HF patients within 2 weeks of discharge. There is particular focus on improving coding, confirming subtype, providing patient education and self-management, providing early HFN review post discharge and follow-up, and ensuring a seamless transition between hospital and community services. | Y | Y | Y | Y | UC | UC | UC | Y | Y | 578 | 81 | 71 | 181 | 31 |
| Monteiro, C. and Cojoianu, A. and Savage, R. and Bone, R. and Hammond, C. and Gamble, J. and Newton, J. | Clinical audit of in-patient echocardiography in acute heart failure: Real world data from a tertiary hospital | 2020 | European Heart Journal Cardiovascular Imaging | abstract | Retrospective observational | Our centre has a specialist team who assesses patients with suspected HF on admission, and refers them for urgent in-patient TTE, using two priority in-patient echo slots per day. Patients are initially referred for HF assessment by general medics and geriatricians, across nonspecialist medical wards. We audited the referrals and results of those who received TTE in this context. | Oxford | ns | ns | nc | ns | UC | ns | ns | ns | ns | ns | ns | ns | ns | Our centre has a specialist team who assesses patients with suspected HF on admission, and refers them for urgent in-patient TTE, using two priority in-patient echo slots per day. Patients are initially referred for HF assessment by general medics and geriatricians, across nonspecialist medical wards. We audited the referrals and results of those who received TTE in this context. | na | na | Y | Y | Y | UC | UC | UC | UC | UC | Y | 252 | 80 | 126 | 126 | 50 |
| Cannata, A. and Badawy, L. and Anyu, A. T. and Samways, J. and Sweeney, M. and Jordan-Rios, A. and Zakeri, R. and Scott, P. A. and Piper, S. and Plymen, C. M. and McDonagh, T. A. and Bromage, D. I. | The prognostic impact of specialist cardiology input in patients admitted for heart failure and normal ejection fraction | 2023 | ESC heart failure | full paper | Prospective cohort study | To describe the association of specialist cardiology inout and outcomes in patients admitted with decompensated HFpEF. | London | ns | ns | nc | ns | UC | ns | ns | ns | ns | ns | ns | ns | ns | Specialist input was classified as whether they received specialist cardiology input. | na | na | Y | Y | Y | UC | UC | UC | UC | UC | Y | 1091 | 79 | 457 | 1091 | 100 |
| Simms, J. and Irani, T. and Schiff, R. | 38 HEART FAILURE IN THE OLDER PERSON: IS THERE STILL A PLACE FOR THE GERIATRICIAN? | 2014 | Age & Ageing | abstract | Retrospective observational | The objective was to survey older patients’ characteristics and management for acute heart failure admission to the Older Persons Care Unit. | London | ns | ns | nc | ns | UC | ns | ns | ns | ns | Geriatricians | ns | ns | ns | We sampled forty-five patients admitted to the Older Persons’ Unit with heart failure ( January-March 2013) collecting data from case notes and discharge summaries, adapting the National Heart Failure Audit tool adding domains including cognition, non-cardiac co-morbidities and dependency. | na | na | Y | Y | Y | UC | UC | UC | UC | UC | Y | 45 | 86 | ns | 28 | 62 |
| Tran, P. and Long, T. and Smith, J. and Kuehl, M. and Mahdy, T. and Banerjee, P. | Developing a contemporary community clinic for patients with heart failure with preserved ejection fraction within the current National Health Service model | 2022 | Open Heart | full paper | Retrospective observational | Evaluation of the efficacy of an existing dedicated HFpEF clinic to find innovative ways to design a more comprehensive model tailored to the modern era of HFpEF. | Coventry & Warwickshire | Consultant led | ns | no | yes | Y | ns | ns | ns | ns | ns | ns | ns | ns | UHCW is a tertiary cardiac centre that runs daily consultant-led general cardiology clinics, where a mixture of patients with HFrEF and HFpEF are seen by both HF and non-HF specialists including trainee registrars. Prior to the global viral pandemic when all consultations were held face to face, a specific HFpEF community clinic was set up, which was solely led by a consultant cardiologist specialising in HF in the City of Coventry Health Centre, UK. | The dedicated HFpEF clinic was a smaller clinic which saw approximately up to 6 suspected HFpEF patients every week as opposed to the general clinic which was a larger clinic of around 24 patients seeing a mixture of HF patients of all types including HFpEF. As the latter clinic was busier, not all patients seen by cardiology registrars in that clinic were necessarily reviewed or discussed with the consultant cardiologist. | Furthermore, the dedicated HFpEF clinic was also involved in recruiting patients into research trials, for example, the earlier PARAGON study. | Y | Y | Y | Y | UC | Y | UC | Y | Y | 101 | 78 | 46 | 101 | 100 |
| Shah, J. and Paz, E. and Elias, E. and Alimo, A. and Sharma, S. and Elizondo, I. and Malone, C. and Milner, P. and Winn, T. | The Challenges and Opportunities of Starting a Heart Failure Virtual Ward, Experience from London North West University Healthcare Nhs Trust | 2022 | Heart | abstract | Retrospective observational | To present an analysis of the first 198 days of the HF teams experience of launching a heart failure virtual ward with a remote monitoring app service that allows patients to upload daily observations and symptoms status to aid in symptom management and optimisation of medical therapy. | London | Virtual HF clinic | ns | no | ns | UC | ns | ns | ns | ns | ns | ns | ns | ns | London North West University Healthcare NHS Trust (LNWUH) partnered with LUSCIITM, a remote monitoring app that allows patients to upload daily observations and symptoms status to aid in symptom management and optimisation of medical therapy. | na | na | Y | Y | Y | UC | UC | UC | UC | Y | Y | 64 | 62 | 48 | 6 | 10 |
| Thompson, A. and Crilley, J. and Wilson, D. and Hungin, A. P. S. and Fuat, A. and Murphy, J. | An Epidemic of HFPEF? | 2016 | Heart | abstract | Prospective cohort study | To differentiate a heart failure population with contemporary diagnostic parameters | County Durhum and Darlington | ns | ns | nc | ns | UC | ns | ns | ns | ns | ns | ns | ns | ns | Described only as a heart failure clinic. | na | na | Y | UC | UC | UC | UC | UC | UC | UC | UC | 245 | ns | ns | 34 | 14 |
| Nguyen, M. and Rumjaun, S. and Lowe-Jones, R. and Ster, I. C. and Rosano, G. and Anderson, L. and Banerjee, D. | Management and outcomes of heart failure patients with CKD: experience from an inter-disciplinary clinic | 2020 | ESC heart failure | abstract | Retrospective observational | To describe the experiences of setting up and running a nvel combined kidney fialure/heart failure clinic and to evaluate its effectivness. | London | Interdisciplinary | ns | nc | yes | Y | ns | ns | ns | ns | nephrologist & anaemic nurse specialist input | ns | ns | ns | Criteiria for referral to the kidney failure-heart failure (KFHF) clinic were concomitant CKD (stage 3 or above) and heart failure. Patients were followed up at varying frequencies as per clinical need and discharged when they are stable on maximally tolerated therapy. | na | na | Y | Y | Y | UC | UC | UC | UC | UC | UC | 124 | 78.5 | ns | 25 | 20 |
| Sinclair, H. and Ackrill, M. and Holdsworth, H. and Chase, C. and Guillen, M. and Bowman, L. and Collins, L. and Critoph, C. H. and Pine, A. C. J. | Rapid access heart failure clinic: Impact of a physiologist-delivered service in a UK district general hospital | 2019 | Heart | abstract | Retrospective observational | To assess the safety and efficacy of a rapid access cardiac physiologist led HF clinic. | Bournemouth | Consultant oversight | Cardiac physiologist led | yes | ns | UC | 1 | ns | 1 | ns | cardiac physiologists | ns | ns | ns | In the Royal Bournemouth hospital, Rapid Access HF Clinic Referrals (RAHFC) referrals have doubled in a year. Initial service evaluation found that only 47% of patients were seen within the target waiting time. It was proposed this lower range NTproBNP population (400- 1000ng/l) could be safely managed in a cardiac physiologist-delivered RAHFC overseen by a HF specialist. Additonal benefits would be to upskill cardiac physiologists, free consultant time for more severe HF patients and improve waiting times. | The clinic was initially run by 2 cardiac physiologists and a specialist HF fellow. All clinics were overseen by a consultant HF cardiologist. | na | Y | UC | UC | UC | UC | UC | UC | UC | UC | 34 | 81 | 41 | 9 | 26.9 |
| Zheng, A. and Cowan, E. and Philip, L. and Guha, K. and Kalra, P. R. and Morton, G. | Characteristics and outcomes of patients with suspected heart failure and elevated natriuretic peptides referred to a nice-compliant heart failure clinic | 2019 | Heart | full paper | Retrospective observational | The aim was to describe patient characteristics, HF diagnostic rates, and 1-year hospitalisation and mortality rates in a multicentre, real-world setting using this NICE model. | Porstmouth & Southampton | Consultant led | ns | no | ns | UC | 1 | ns | 1 | ns | cardiographer & cardiac physiologist | community HF nurse team | ns | ns | A dedicated one-stop HF clinic that involves all patients undergoing an ECG and echocardiography by a cardiographer and cardiac physiologist, respectively, and specialist assessment by a physician, during a single visit. dedicated HF clinics for specialist (HF consultant or a supervised senior HF trainee) assessment and echocardiography, in line with the contemporary NICE guidelines. | Following clinical assessment, ECG and echocardiography, a diagnosis of HF was either confirmed or excluded by a HF specialist. HF was subdivided into HF with reduced ejection fraction (HFrEF) or HF with preserved ejection fraction (HFpEF) based on accepted definitions and the clinical decision of the HF specialist. Patients with HFrEF routinely received ongoing follow-up from a multidisciplinary team, mainly from community HF specialist nurses supported by HF specialists. Those with HFpEF and those without HF (NHF) were usually discharged to primary care with a diagnosis and management plan where possible. |  | Y | Y | Y | UC | UC | UC | UC | Y | Y | 1271 | 80 | 591 | 329 | 26 |
| Horan, C. and Bower, C. and Kennedy, J. and O'Pray, A. and Crilly, M. and Thronton-Clay, H. and Riley, M. and Brown, K. and Balu, A. and Oguguo, E. and Sankaranarayanan, R. | Outcomes of heart failure specialist nurse delivered consultant led community virtual heart failure multidisciplinary team meetings during the first peak of the COVID-19 pandemic | 2021 | European Journal of Heart Failure | abstract | Retrospective observational | To compare the outcomes of a virtual community HF MDT versus standard (pre-covid) HF MDT clinics | Liverpool | Consultant oversight | Nurse led | yes | ns | UC | ns | ns | ns | ns | ns | ns | Palliative care | ns | Virtual community HF clinic MDTs conducted by HF speciliaist nurese under the leadership of a HF Consultant. | na | na | Y | Y | Y | UC | UC | UC | UC | Y | Y | 181 | 79.1 | ns | 90 | 50 |
| Doleman, F. and Santon, M. and Clewes, J. and Laithwaite, C. and McIntosh, R. and Ahmed, H. and Ahmed, N. | Evaluation of an ambulatory heart failure service-a 2 years' experience | 2019 | European Heart Journal | abstract | Retrospective observational | To review the efficacy of an Nurse-led ambulatory heart failure unit | Derby | ns | Nurse led | nc | ns | UC | ns | ns | ns | ns | ns | ns | ns | ns | An ambulatory HF unit, run by specalist nurses that seeks to reduce admission to hospital for HF by administering intravenous diuretics in a day unit setting for ambulatory patients. | na | na | Y | UC | Y | UC | UC | UC | UC | UC | UC | 393 | ns | ns | 144 | 37 |
| Al-Mohammad, A. and Watt, V. and O'Toole, L. and Hall, I. and Yates, L. | Insights into the epidemiology of incident Heart Failure (HF): Outcomes of rapid HF access clinic applying the NICE guidelines | 2013 | European Heart Journal | abstract | Retrospective observational | To report the acitvity of a newly established rapid access, one stop shop heart failrue clinic. | Sheffield | Consultant led | ns | no | ns | UC | ns | ns | ns | ns | ns | ns | ns | ns | Establisjed 3-weekly clinics with the capacity of 18 pateints per week. Patients underwent ECG and Echo on the same day of the clinic visit. In the first 9 months of the clinic, the tea, accepted 605 referrals, of which 539 attended the clinic. | na | na | Y | Y | Y | UC | UC | UC | UC | UC | UC | 539 | ns | ns | 196 | 36 |
| Dulai, R. and Sheikh, A. S. and Qureshi, A. and Katechia, S. and Peysakhova, Y. and Johns, M. and Mazhar, S. | Prevalence, clinical characteristics and outcomes of HF with preserved versus reduced ejection fraction | 2016 | British Journal of Cardiology | full paper | Retrospective observational | The main aim of our study was to assess the characteristics, treatment and short-term outcome of patients with HFPEF following admission with acute decompensated heart failure compared with those patients with HFREF | Southend | ns | ns | ns | ns | UC | ns | ns | ns | ns | ns | ns | ns | ns | In-patient service with community based follow-up for HFrEF. | na | na | Y | UC | Y | UC | UC | UC | UC | Y | UC | 241 | 84 | 12 | 41 | 17 |
| Helena Bolam, H. and Kalra, P. R. and Guha, K. and Morton, G. D. J. | The impact of a clinical educational and self-care intervention in patients with heart failure with preserved ejection fraction | 2019 | European Journal of Heart Failure | abstract | Prospective cohort study | To assess the impact of a 'HFpEF extenson clinic' that provided clinical educational and selfcare interventions, on outcomes in patients diagnosed with HFpEF. | Portsmouth | ns | ns | ns | ns | UC | ns | ns | ns | ns | ns | ns | ns | ns | 25 consecutive patients newly diagnosed with HFpEF were offered an additional comprehensive individualised intervention including clinical review, risk factor modification, education and selfmanagement training via a 'HFpEF extension clinic' that is normally only avalable to HFrEF. | na | na | Y | Y | Y | Y | UC | UC | UC | UC | UC | 25 | 83 | 8 | 32 | 25 |
| Griffiths, A. and Taylor, J. and McRae, D. | Evaluation of a specialist pharmacist led diagnostic chronic heart failure clinic | 2020 |  | poster | Retrospective observational | To describe the development, implementation, and evaluation of pharmacist-led heart failure clinics with respect to time from referral to diagnosis, time from diagnosis to frst review with a specialist, and the proportion receiving optimal GDMT 180 days after diagnosis. | Hywel Dda University Health Board | Consultant oversight | Pharmacist led | Y | Y | Y | ns | ns | ns | yes | ns | Cardiac imaging | ns | ns | All referrals from GPs for suspected HF were triaged by consultant cardiologists to one of three weekly diagnostic clinics. NT-pro BNP measurement was widely available in primary care and used, as per NICE guidance, to stratify patients to ‘Urgent’ (NT-pro BNP>2,000 ng/L, ideally attend within 2 weeks) and ‘Routine’ (NT-pro BNP between 400–2000 ng/L, ideally attend within 6 weeks). At clinic, a healthcare support worker recorded blood pressure, pulse rate, respiratory rate and oxygen saturation, an ECG was performed by a cardiac physiologist and a detailed echocardiogram by experienced physiologist/sonographer. | The lead pharmacist completed a clinical consultation (receiving the history, performing a physical examination, and reviewing and interpreting the blood analyses, ECG and echocardiogram). Where appropriate a formal diagnosis of HF was made by the lead pharmacist who then initiated GDMT at that point. | Patients with HFrEF then attended a follow-up clinic provided by another pharmacist. The purpose of this second clinic (co-located and running concurrently with the frst to allow peer-support) was to pursue and monitor the rapid optimisation of GDMT as per the plan formulated at the diagnostic clinic. At the frst review with the advanced pharmacist, patients elected either to attend further face-to-face appointments or are enrolled to a remote telehealth service. In the latter case patients were given, and received tuition to enable use of, equipment to measure daily blood pressure, weight, pulse rate, and oxygen saturation using an iPad link via Bluetooth. Weekly or fortnightly telephone consultations of these patients were undertaken by the advanced pharmacist, with relevant prescriptions and blood analysis request forms posted to patients for dispensing at their local community pharmacy and blood testing at their GP surgery. | Y | Y | Y | Y | UC | UC | Y | Y | Y | 111 | ns | ns | ns | ns |
| Garg, P. Dakshi, A. Assadi, H. Swift, AJ. Naveed, U. Fent, G. Lewis, Rogers, D.  Charalampopoulos, A. Al-Mohammad A. | Characterisation of the patients with suspected heart failure: experience from the SHEAF registry | 2021 | Open Heart | full paper | Prospective cohort study | To characterise and risk-stratify patients presenting to a heart failure (HF) clinic according to the National Institute for health and Care Excellence (NICE) algorithm. | Sheffield | ns | ns | ns | ns | UC | ns | ns | ns | ns | ns | ns | ns | ns | All patients referred to the HF clinic by their GPs with NT-proBNP >400 pg/mL underwent a resting 12-lead ECG and TTE. Each patient was clinically assessed by a specialist. The final diagnosis was determined by the HF specialist integrating the presenting history, clinical examination and the results of investigations in keeping with NICE guidelines. | na | na | Y | UC | UC | UC | UC | UC | UC | UC | UC | 4368 | 80 | 1999 | 2022 | 46.2 |
| Morton, G. Philip, L. Gilpin, T. Chan, PR. Guha, K. Kalra, PR. | Does specialist review for patients with suspected heart failure predict better outcomes? An observational study on the utility of compliance with NICE guidelines | 2018 | BMJ Open | full paper | Retrospective observational | To compare outcomes in patients with suspected HF and raised natriuretic peptides who are reviewed in a specialist HF clinic in line with National Institute for Health and Care Excellence (NICE) guideline, versus patients who are not reviewed in. | Portsmouth | Consultant led | ns | ns | ns | UC | ns | ns | ns | ns | ns | ns | ns | ns | Following the publication of the 2010 NICE chronic HF guidelines,a dedicated one-stop, fully NICE compliant referral pathway and specialist clinic was established at our institution to assess patients with suspected HF. The clinic launch was accompanied by education of referring general practitioners (GPs) and provision of NT-pro-BNP assays. | Referrals to the specialist HF clinic were sent via a dedicated fax number. Patients referred with an NT-pro-BNP of >2000 pg/mL were seen within 2 weeks and those with an NT-pro-BNP of 400–2000 pg/mL within 6 weeks. Participants were seen in a one-stop clinic and received specialist assessment and echocardiography. A diagnosis of HF could then be confirmed or excluded, and an appropriate management plan formulated. | European Society of Cardiology (ESC) HF guidelines10 wherever possible and appropriate. Those with HFREF routinely received early and ongoing management from a multidisciplinary team centred around community-based HF nurse specialists and supported by HF specialists. Those with a diagnosis of HFPEF and those without HF were usually discharged back to the GP with a management plan. | Y | Y | Y | UC | UC | UC | UC | UC | UC | 161 | 78 | 78 | 46 | 54 |
| Peplow & Rees | An evaluation of a service expansion to include patients with heart failure with preserved ejection fraction | 2024 | British Journal of Cardiac Nursing | full paper | Retrospective observational | To evaluate the impact of service expansion on service referral rates, length of stay in the service and clinical workload | West Hertforshire | Consultant led | Nurse led | Y | Y | Y | 1 | 1 | ns | ns | Y | social care | na | na | The new patients with heart failure with preserved ejection fraction were admitted to the service in May 2020. The expectation on planning this service was that a management plan would be put in place for the patients with heart failure with preserved ejection fraction by a consultant cardiologist, and a heart failure specialist nurse would be appointed to implement this. | The plan was for only one appointment for each patient to be made. In reality, this aim was found to be unrealistic because of a combination of reasons, including comorbidities, inadequate team−team referrals and poor social support. Resources were not adjusted to account for this, and no revised targets or expectations were made. Despite improved processes, the service has struggled to manage referrals. | There were a greater number of multidisciplinary team or consultant reviews in the heart failure with preserved ejection fraction cohort, suggesting that they are the more complex heart failure type when it comes to management, and are more likely to require time-consuming reviews. This may be exacerbated by the lack of treatment  guidance for this group. | Y | Y | Y | Y | UC | UC | Y | Y | Y | 81 | 85 | ns | 40 | 49% |
| Tavares et al. | Characteristics and outcomes of patients with heart failure with preserved ejection fraction referred to a community specialist nurse-led clinic | 2024 | British Journal of Cardiac Nursing | full paper | Prospective cohort study | To report on the components of current specialist care and to understand the characteristics of service users with HFpEF. | North West London | Consultant led | Nurse led | Y | Y | Y | UC | UC | UC | UC | Y | renal medicine | palliative care | clinical psychologist | The service was established in 2016 in north west London and accepts all patients with suspected or confirmed heart failure. Referrals must adhere to The National Institute for Health and Care Excellence (2018) guidance for the diagnosis and management of heart failure in adults. Natriuretic peptide laboratory results inform scheduling of a transthoracic echocardiography in the absence of previous cardiac imaging. A heart failure specialist nurse or cardiology consultant then confirms the diagnosis of HFpEF (Table 1) by reviewing the echocardiography report, based on the current European Society of Cardiology guidelines. | The wider multidisciplinary team includes consultants in cardiology, renal medicine and palliative care, as well as a clinical psychologist. This collaborative approach ensures comprehensive care, particularly for complex cases or patients with advanced care needs. The clinic can refer patients to local cardiac rehabilitation teams for additional support, including access to dieticians, exercise specialists and cardiac rehabilitation nurses. The heart failure specialist nurses manage a caseload of 50–60 patients each. They are independent non-medical prescribers (Royal Pharmaceutical Society, 2021), with advanced skills that are aligned with the British Society for Health Failure’s heart failure specialist nurse competency framework. | A core aspect of heart failure specialist nursing care is encouraging health-promoting behaviours through education on self-care. This includes educating patients on their HFpEF diagnosis, how to recognise signs of fluid overload and the need for regular weight monitoring. Additionally, patients are instructed on medication adherence and potential adverse effects, as well as the importance of avoiding excess salt. Diagnostic tests, such as echocardiograms and electrocardiograms, are reviewed during consultations and referrals to multidisciplinary services are made when necessary. Once a patient’s fluid status is optimised and stabilised, they may be discharged to their primary care physician or placed on a patient-initiated follow-up pathway. Under this pathway, patients receive yearly reviews from a consultant cardiologist to ensure access to ongoing management and support. | Y | Y | Y | Y | UC | Y | Y | Y | Y | 80 | 82 | 31 | 80 | 100% |
| Migas et al | Missed opportunities in heart failure diagnosis and management: study of an urban UK population | 2024 | ESC Heart Failure | full paper | Retrospective observational | to examine the diagnostic pathways and outcomes of patients with heart failure (HF), stratified by left ventricular ejection fraction (EF), and to highlight deficiencies in real-world HF diagnosis and management. | Salford | Consultant led | UC | Y | UC | Y | UC | UC | UC | UC | Y | cardio-respiratory | na | na | Cardiology-related outpatient clinics included cardiology (45%), HF (17%), and cardio-respiratory investigations (38%). In total, 65.1% of the HF cohort had utilized a cardiology-led outpatient clinic prior to the diagnosis, increasing to 77.1% post-diagnosis. Specifically, within the first 12 months following HF diagnosis, overall, 72.5% of patients had at least one specialist-led outpatient visit; for HFpEF, this was 78.7% of patients. | na | na | Y | Y | Y | UC | UC | Y | UC | UC | Y | 3227 | 78 | 702 | 1505 | 46.60% |
| Murphy et al. | Can the use of a structured management approach using ABCDEF mnemonic to manage multi-morbdiity in HFpEF improve clinical outcomes? | 2024 | Heart | abstract | Prospective cohort study | To test the use of a structured approach by HF specialist nurse using the prompt of an ABCDEF mnemonic to guide management of multimorbidity in HFpEF clinics and compare this with standard care (without prompt by a mnemonic). | Liverpool | Consultant oversight | UC | UC | UC | Y | UC | UC | UC | UC | UC | ns | na | na | Patients were reviewed in a clinic by a HF specialist nurse. (38 patients in the "mnemonic" cohort and 37 patients in the standard care cohort). ABCDEF mnemonic was used to prompt the HFspecialist nurse to address co-morbidities in the "mnemonic cohort" (A-Anaemia/Atrial fibrillation/sleep Apnoea, B- Blood pressure control, Body Mass Index, C- Chronic Kidney Disease and Chronic Obstructive Pulmonary disease, D- Diabetes control, E- Exercise rehab, F- Frailty assessment and need for advance care planning) | na | n | Y | Y | Y | Y | Y | Y | UC | UC | UC | 38 | 77.5 | 17 | 38 | 100.00% |

## Table 1: Clinical service description

| **Authors** | **Title** | **Year** | **Sub-type** | **Aim** | **Clinic Location** | **Description of Clinic Types 1** | **Description of Clinic Types 2** | **MDT** | **Service links** |
| --- | --- | --- | --- | --- | --- | --- | --- | --- | --- |
| Guha *et al.^1^* | Audit of a tertiary heart failure outpatient service to assess compliance with NICE guidelines | 2016 | Retrospective observational / audit | To compare 2013 audit data to 2011 audit data on admissions to a tertiary cardiac centre. | London | Consultant led | Nurse led | Yes | Cardiac imaging, palliative care |
| Murphy *et al.^2^* | A Comparison of HFrEF Vs HFpEF Clinical Workload and Cost in the First Year Following Hospitalisation and Enrolment in a Disease Management Program | 2017 | Retrospective observational | To investigate resource utilization and cost comparing heart failure with reduced ejection fraction and HFpEF. | Dublin | Consultant led | Nurse led | Yes | Not stated |
| Hassan *et al.^3^* | Heart failure specialist nurse-led day case ambulatory management with intravenous diuretics reduces hospitalisations for acute decompensated heart failure irrespective of ejection fraction | 2018 | Retrospective observational | To describe the development of a nurse led ambulatory HF unit. | Liverpool | Consultant oversight | Nurse led | Yes | Renal, Pleural / ascites specialist, Palliative care |
| Hawley *et al.^4^* | The impact of an integrated heart failure service in a medium-sized district general hospital | 2020 | Retrospective observational | To assess the impact of a new integrated heart failure service (IHFS) in a medium-sized district general hospital (DGH) on heart failure (HF) mortality, readmission rates, and provision of HF care. | Torbay & South Devon | Consultant led | Nurse led | Yes | Renal, palliative, care of the elderly |
| Monteiro *et al.^5^* | Clinical audit of in-patient echocardiography in acute heart failure: Real world data from a tertiary hospital | 2020 | Retrospective observational | Our centre has a specialist team who assesses patients with suspected HF on admission, and refers them for urgent in-patient TTE, using two priority in-patient echo slots per day. Patients are initially referred for HF assessment by general medics and geriatricians, across nonspecialist medical wards. We audited the referrals and results of those who received treatment in this context. | Oxford | Not stated | Not stated | Not stated | Not stated |
| Cannata *et al.^6^* | The prognostic impact of specialist cardiology input in patients admitted for heart failure and normal ejection fraction | 2023 | Prospective cohort study | To describe the association of specialist cardiology input and outcomes in patients admitted with decompensated HFpEF. | London | Not stated | Not stated | Not stated | Not stated |
| Simms *et al.^7^* | HEART FAILURE IN THE OLDER PERSON: IS THERE STILL A PLACE FOR THE GERIATRICIAN? | 2014 | Retrospective observational | The objective was to survey older patients’ characteristics and management for acute heart failure admission to the Older Persons Care Unit. | London | Not stated | Not stated | Not stated | Not stated |
| Tran *et al.^8^* | Developing a contemporary community clinic for patients with heart failure with preserved ejection fraction within the current National Health Service model | 2022 | Retrospective observational | Evaluation of the efficacy of an existing dedicated HFpEF clinic to find innovative ways to design a more comprehensive model tailored to the modern era of HFpEF. | Coventry & Warwickshire | Consultant led | Not stated | Yes | Not stated |
| Shah *et al.^9^* | The Challenges and Opportunities of Starting a Heart Failure Virtual Ward, Experience from London North West University Healthcare Nhs Trust | 2022 | Retrospective observational | To present an analysis of the first 198 days of the HF teams experience of launching a heart failure virtual ward with a remote monitoring app service that allows patients to upload daily observations and symptoms status to aid in symptom management and optimisation of medical therapy. | London | Virtual HF clinic | Not stated | Not stated | Not stated |
| Thompson *et al.^10^* | An Epidemic of HFPEF? | 2016 | Prospective cohort study | To differentiate a heart failure population with contemporary diagnostic parameters | County Durham and Darlington | Not stated | Not stated | Not stated | Not stated |
| Nguyen *et al.^11^* | Management and outcomes of heart failure patients with CKD: experience from an inter-disciplinary clinic | 2020 | Retrospective observational | To describe the experiences of setting up and running a novel combined kidney failure/heart failure clinic and to evaluate its effectiveness. | London | Interdisciplinary | Not stated | Yes | Not stated |
| Sinclair *et al.^12^* | Rapid access heart failure clinic: Impact of a physiologist-delivered service in a UK district general hospital | 2019 | Retrospective observational | To assess the safety and efficacy of a rapid access cardiac physiologist led HF clinic. | Bournemouth | Consultant oversight | Cardiac physiologist led | Not stated | Not stated |
| Zheng *et al.^13^* | Characteristics and outcomes of patients with suspected heart failure and elevated natriuretic peptides referred to a nice-compliant heart failure clinic | 2019 | Retrospective observational | The aim was to describe patient characteristics, HF diagnostic rates, and 1-year hospitalisation and mortality rates in a multicentre, real-world setting using this NICE model. | Portsmouth & Southampton | Consultant led | Not stated | Not stated | community HF nurse team |
| Horan *et al.^14^* | Outcomes of heart failure specialist nurse delivered consultant led community virtual heart failure multidisciplinary team meetings during the first peak of the COVID-19 pandemic | 2021 | Retrospective observational | To compare the outcomes of a virtual community HF MDT versus standard (pre-covid) HF MDT clinics | Liverpool | Consultant oversight | Nurse led | Not stated | Palliative care |
| Doleman *et al.^15^* | Evaluation of an ambulatory heart failure service-a 2 years' experience | 2019 | Retrospective observational | To review the efficacy of an Nurse-led ambulatory heart failure unit | Derby | Not stated | Nurse led | Not stated | Not stated |
| Al-Mohammad *et al.^16^* | Insights into the epidemiology of incident Heart Failure (HF): Outcomes of rapid HF access clinic applying the NICE guidelines | 2013 | Retrospective observational | To report the activity of a newly established rapid access, one stop shop heart failure clinic. | Sheffield | Consultant led | Not stated | Not stated | Not stated |
| Dulai *et al.^17^* | Prevalence, clinical characteristics and outcomes of HF with preserved versus reduced ejection fraction | 2016 | Retrospective observational | The main aim of our study was to assess the characteristics, treatment and short-term outcome of patients with HFPEF following admission with acute decompensated heart failure compared with those patients with HFREF | Southend | Not stated | Not stated | Not stated | Not stated |
| Bolam *et al.^18^* | The impact of a clinical educational and self-care intervention in patients with heart failure with preserved ejection fraction | 2019 | Prospective cohort study | To assess the impact of a 'HFpEF extension clinic' that provided clinical educational and selfcare interventions, on outcomes in patients diagnosed with HFpEF. | Portsmouth | Not stated | Not stated | Not stated | Not stated |
| Griffiths *et al.^19^* | Evaluation of a specialist pharmacist led diagnostic chronic heart failure clinic | 2020 | Retrospective observational | To describe the development, implementation, and evaluation of pharmacist-led heart failure clinics with respect to time from referral to diagnosis, time from diagnosis to first review with a specialist, and the proportion receiving optimal GDMT 180 days after diagnosis. | Hywel Dda University Health Board | Consultant oversight | Pharmacist led | Yes | Cardiac imaging |
| Garg *et al.^20^* | Characterisation of the patients with suspected heart failure: experience from the SHEAF registry | 2021 | Prospective cohort study | To characterise and risk-stratify patients presenting to a heart failure (HF) clinic according to the National Institute for health and Care Excellence (NICE) algorithm. | Sheffield | Not stated | Not stated | Not stated | Not stated |
| Morton *et al.^21^* | Does specialist review for patients with suspected heart failure predict better outcomes? An observational study on the utility of compliance with NICE guidelines | 2018 | Retrospective observational | To compare outcomes in patients with suspected HF and raised natriuretic peptides who are reviewed in a specialist HF clinic in line with National Institute for Health and Care Excellence (NICE) guideline, versus patients who are not reviewed in. | Portsmouth | Consultant led | Not stated | Not stated | Not stated |
| Peplow & Rees^22^ | An evaluation of a service expansion to include patients with heart failure with preserved ejection fraction | 2024 | Retrospective observational | To evaluate the impact of service expansion on service referral rates, length of stay in the service and clinical workload | West Hertfordshire | Consultant led | Nurse led | Yes | social care |
| Tavares *et al.^23^* | Characteristics and outcomes of patients with heart failure with preserved ejection fraction referred to a community specialist nurse-led clinic | 2024 | Prospective cohort study | To report on the components of current specialist care and to understand the characteristics of service users with HFpEF. | North West London | Consultant led | Nurse led | Yes | renal medicine, palliative care, clinical psychology |
| Migas *et al.^24^* | Missed opportunities in heart failure diagnosis and management: study of an urban UK population | 2024 | Retrospective observational | to examine the diagnostic pathways and outcomes of patients with heart failure (HF), stratified by left ventricular ejection fraction (EF), and to highlight deficiencies in real-world HF diagnosis and management. | Salford | Consultant led | Not stated | Not stated | cardio-respiratory |
| Murphy *et al.^25^* | Can the use of a structured management approach using ABCDEF mnemonic to manage multi-morbidity in HFpEF improve clinical outcomes? | 2024 | Prospective cohort study | To test the use of a structured approach by HF specialist nurse using the prompt of an ABCDEF mnemonic to guide management of multimorbidity in HFpEF clinics and compare this with standard care (without prompt by a mnemonic). | Liverpool | Consultant oversight | Not stated | Not stated | Not stated |

## Table 2: Clinical service / management characteristics

| **Authors** | **clinical assessment** | **cardiological optimisation** | **medication optimisation** | **patient education** | **functional assessment** | **rehab referral** | **telephone triage** | **community outreach / review** | **routine follow-up** |
| --- | --- | --- | --- | --- | --- | --- | --- | --- | --- |
| Guha *et al.^1^* | Y | Y | Y | Y | Y | Y | UC | UC | Y |
| Murphy *et al.^2^* | Y | Y | Y | Y | UC | UC | Y | UC | Y |
| Hassan *et al.^3^* | Y | Y | UC | UC | UC | UC | UC | UC | UC |
| Hawley *et al.^4^* | Y | Y | Y | Y | UC | UC | UC | Y | Y |
| Monteiro *et al.^5^* | Y | Y | Y | UC | UC | UC | UC | UC | Y |
| Cannata *et al.^6^* | Y | Y | Y | UC | UC | UC | UC | UC | Y |
| Simms *et al.^7^* | Y | Y | Y | UC | UC | UC | UC | UC | Y |
| Tran *et al.^26^* | Y | Y | Y | Y | UC | Y | UC | Y | Y |
| Shah *et al.^9^* | Y | Y | Y | UC | UC | UC | UC | Y | Y |
| Thompson *et al.^10^* | Y | UC | UC | UC | UC | UC | UC | UC | UC |
| Nguyen *et al.^11^* | Y | Y | Y | UC | UC | UC | UC | UC | UC |
| Sinclair *et al.^12^* | Y | UC | UC | UC | UC | UC | UC | UC | UC |
| Zheng *et al.^13^* | Y | Y | Y | UC | UC | UC | UC | Y | Y |
| Horan *et al.^14^* | Y | Y | Y | UC | UC | UC | UC | Y | Y |
| Doleman *et al.^15^* | Y | UC | Y | UC | UC | UC | UC | UC | UC |
| Al-Mohammad *et al.^16^* | Y | Y | Y | UC | UC | UC | UC | UC | UC |
| Dulai *et al.^17^* | Y | UC | Y | UC | UC | UC | UC | Y | UC |
| Bolam *et al.^18^* | Y | Y | Y | Y | UC | UC | UC | UC | UC |
| Griffiths et a. | Y | Y | Y | Y | UC | UC | Y | Y | Y |
| Garg *et al.^20^* | Y | UC | UC | UC | UC | UC | UC | UC | UC |
| Morton *et al.^21^* | Y | Y | Y | UC | UC | UC | UC | UC | UC |
| Peplow & Rees^22^ | Y | Y | Y | Y | UC | UC | Y | Y | Y |
| Tavares *et al.^23^* | Y | Y | Y | Y | UC | Y | Y | Y | Y |
| Migas *et al.^24^* | Y | Y | Y | UC | UC | Y | UC | UC | Y |
| Murphy *et al.^25^* | Y | Y | Y | Y | Y | Y | UC | UC | UC |

## Table 3: Sample characteristics

| **Authors** | **Sample (n)** | **Age (all, years)** | **Sex (male)** | **HFpEF (n)** | **HFpEF**  **(%)** | **NYHA Class** | **NTpro BNP** | **BNP** | **Charlson comorbidity index** | **Cognitive assessment** | **cardiac referral** | **Rehab referral (n)** |
| --- | --- | --- | --- | --- | --- | --- | --- | --- | --- | --- | --- | --- |
| Guha *et al.^1^* | 500 | 68 | 352 | 95 | 19 | ns | ns | ns | ns | ns | y | 15 |
| Murphy *et al.^2^* | 1292 | 74.5 | 759 | 413 | 32 | IV | ns | 602 | 7 | ns | ns | ns |
| Hassan *et al.^3^* | 335 | 80 | ns | 117 | 35 | ns | ns | 2057 | ns | ns | ns | ns |
| Hawley *et al.^4^* | 578 | 81 | 71 | 181 | 31 | IV | ns | ns | ns | ns | ns | ns |
| Monteiro *et al.^5^* | 252 | 80 | 126 | 126 | 50 | ns | ns | ns | ns | ns | ns | ns |
| Cannata *et al.^6^* | 1091 | 79 | 457 | 1091 | 100 | III/IV | ns | ns | ns | ns | ns | ns |
| Simms *et al.^7^* | 45 | 86 | ns | 28 | 62 | ns | ns | ns | ns | 32% cognitive impairment | ns | ns |
| Tran *et al.^26^* | 101 | 78 | 46 | 101 | 100 | II/III | 367 | ns | ns | ns | ns | ns |
| Shah *et al.^9^* | 64 | 62 | 48 | 6 | 10 | ns | ns | ns | ns | ns | ns | ns |
| Thompson *et al.^10^* | 245 | ns | ns | 34 | 14 | ns | ns | ns | ns | ns | ns | ns |
| Nguyen *et al.^11^* | 124 | 78.5 | ns | 25 | 20 | ns | ns | ns | ns | ns | ns | ns |
| Sinclair *et al.^12^* | 34 | 81 | 41 | 9 | 26.9 | ns | 730 | ns | ns | ns | ns | ns |
| Zheng *et al.^13^* | 1271 | 80 | 591 | 329 | 26 | ns | 1851 | ns | ns | ns | ns | ns |
| Horan *et al.^14^* | 181 | 79.1 | ns | 90 | 50 | ns | ns | ns | ns | ns | ns | ns |
| Doleman *et al.^15^* | 393 | ns | ns | 144 | 37 | ns | ns | ns | ns | ns | ns | ns |
| Al-Mohammad *et al.^16^* | 539 | ns | ns | 196 | 36 | ns | ns | ns | ns | ns | ns | ns |
| Dulai *et al.^17^* | 241 | 84 | 12 | 41 | 17 | ns | ns | ns | ns | ns | ns | ns |
| Bolam *et al.^18^* | 25 | 83 | 8 | 32 | 25 | 100 | ns | ns | ns | ns | ns | ns |
| Griffiths et a. | 111 | ns | ns | ns | ns | ns | ns | ns | ns | ns | ns | ns |
| Garg *et al.^20^* | 4368 | 80 | 1999 | 2022 | 46.2 | ns | 1780 | ns | ns | ns | ns | ns |
| Morton *et al.^21^* | 161 | 78 | 78 | 46 | 54 | ns | ns | ns | ns | ns | ns | ns |
| Peplow & Rees^22^ | 81 | 85 | ns | 40 | 49% | 2 | ns | ns | ns | ns | ns | ns |
| Tavares *et al.^23^* | 80 | 82 | 31 | 80 | 100% | majority NYHA II or III | 1965 | ns | 3 | ns | Y | ns |
| Migas *et al.^24^* | 3227 | 78 | 702 | 1505 | 46.60% | majority NYHA II or III | ns | ns | ns | ns | Y | 3227 |
| Murphy *et al.^25^* | 38 | 77.5 | 17 | 38 | 100.00% | ns | 1590 | ns | ns | ns | ns | ns |

ns = not stated

## Table 4: Operations documents description

| **Authors** | **Titles** | **Year** | **Aim** |
| --- | --- | --- | --- |
| Kings Health Partners^27^ | Heart Failure Specialist Nurse Service Operational Procedures Acute and Community Setting | 2017 | This document provides a guide to the core operational processes required to be followed by the Heart Failure Specialist Nurses (HFSNs) working at King’s College Hospital NHS Foundation Trust, Guy’s and St Thomas’ Hospital NHS Foundation Trust and the community HFSNs (employed by Guy’s and St Thomas’ but serving both Trusts). |
| Imperial College Healthcare^28^ | Chronic Heart Failure Clinical Guide | 2023 | Clinical guide for all departments, including outpatients and community clinics and local GP’s linked to Imperial College Healthcare NHS Trust |
| Sussex Health and Care Partners^29^ | Heart Failure Diagnostic and Treatment Pathways in Primary Care | 2021 | To provide a consolidation of the heart failure diagnostic and treatment pathways: 1) If suspected unconfirmed heart failure then use the diagnostic pathway 2) If heart failure already confirmed use treatment pathway with echo report attached. 3) If referral is for an exacerbation of known heart failure, consider referral back to the heart failure nurse where it meets their criteria or otherwise hospital or community heart failure specialist clinic. |
| Oxford University Hospitals^30^ | Heart Failure Guidance Summary for GPs | 2021 | To provide a top-line summary of the guidance written for use in primary care. |
| University Hospitals of North Midlands^31^ | Standard Operating Procedures for the Ambulatory Heart Failure Units | 2020 | The purpose of these SOPs is to define and formalise some of the tasks that Ambulatory Heart Failure Unit staff have to perform in relation to patients treated within the units. |
| Ayrshire and Arran^32^ | Developing a Pathway for Individuals with HFpEF - Project Evaluation | 2024 | In 2021, NHS Ayrshire and Arran (NHS A&A) was successful in attaining funding from the Scottish Government to develop and pilot an MDT pathway for individuals with HFpEF. The project ran until March 2024, when funding was discontinued. This evaluation described the development and outcomes of the project. |
| Wiltshire Health and Care^33^ | Standard Operational Procedure (SOP) Community Heart Failure Service Ops | 2024 | To provide a definition of a Heart Failure patient eligible for treatment within the Community Heart Failure Service. The document outlines the patient pathway from referral and diagnosis through to discharge. The content supports the operational running of the Community Heart Failure Service and management of the clinic waitlists. |
| Leicestershire Partnership^34^ | Standard operating procedure: - Heart Failure Specialist Nursing Service. Leicestershire Partnership NHS Trust | 2024 | To provide a clear written procedure for staff to understand the community Heart Failure Specialist Nursing Service and its processes. It will include information about the patient pathway from the point of referral to discharge and key information about the roles and responsibilities within the service. |
| West Hertfordshire^35^ | West Hertfordshire Proposed model for Heart failure nurse service West Herts | 2019 | To describe the service and current need for expansion. |

## Table 5: Operations documents clinical service / management 1

| **Authors** | **Clinic Location** | **MDT input / approach** | **HF OR Cardiology Consultant** | **Consultants (n)** | **HFSN (n)** | **Medical trainees** | **Pharmacist** | **Other** | **Service links** | **Service links** | **Service links** |
| --- | --- | --- | --- | --- | --- | --- | --- | --- | --- | --- | --- |
| Kings Health Partners^27^ | London | Y | Y | UC | UC | UC | UC | Y | psychological support | palliative care | UC |
| Imperial College Healthcare^28^ | London | Y | Y | 2 | Y | UC | Y | Y | cardiac physiologist | palliative care | UC |
| Sussex Health and Care Partners^29^ | Sussex | Y | Y | UC | Y | UC | UC | Y | palliative | frailty team | community nursing team |
| Oxford University Hospitals^30^ | Oxford | UC | Y | UC | UC | UC | UC | UC | UC | UC | UC |
| University Hospitals of North Midlands^31^ | Midlands | UC | Y | UC | UC | UC | UC | UC | mental health team | palliative care | UC |
| Ayrshire and Arran^32^ | Ayrshire | Y | UC | UC | 2 | UC | Y | Y | Specialist Physiotherapist | UC | UC |
| Wiltshire Health and Care^33^ | Wiltshire | Y | UC | UC | UC | UC | UC | Y | cardiac physiologist | admin support | Community health services |
| Leicestershire Partnership^34^ | Leicester | Y | UC | UC | Y | UC | UC | Y | palliative | UC | UC |
| West Hertfordshire^35^ | West Herts | Y | Y | 1 | 6 | UC | UC | Y | palliative | UC | UC |

## Table 6: Operations documents clinical service / management 2

| **Authors** | **Clinic Location** | **clinical assessment** | **cardiological optimisation** | **medication optimisation** | **patient education** | **functional assessment** | **rehab referral** | **telephone triage** | **community outreach / review** | **routine follow-up** |
| --- | --- | --- | --- | --- | --- | --- | --- | --- | --- | --- |
| Kings Health Partners^27^ | London | Y | Y | Y | Y | UC | Y | Y | Y | Y |
| Imperial College Healthcare^28^ | London | Y | Y | Y | Y | UC | Y | UC | Y | Y |
| Sussex Health and Care Partners^29^ | Sussex | Y | Y | Y | Y | UC | Y | UC | Y | Y |
| Oxford University Hospitals^30^ | Oxford | Y | Y | Y | UC | UC | UC | UC | UC | Y |
| University Hospitals of North Midlands^31^ | Midlands | Y | Y | Y | Y | UC | Y | Y | UC | Y |
| Ayrshire and Arran^32^ | Ayrshire | Y | Y | Y | Y | UC | y | UC | Y | Y |
| Wiltshire Health and Care^33^ | Wiltshire | Y | Y | Y | Y | UC | Y | UC | Y | Y |
| Leicestershire Partnership^34^ | Leicester | Y | Y | Y | Y | Y | Y | Y | Y | Y |
| West Hertfordshire^35^ | West Herts | Y | Y | Y | Y | UC | Y | UC | Y | Y |

## Table 7 Critical Appraisal Assessment

| **Authors** | **Title** | **1. address a clearly focussed issue?** | **2. recruited in an acceptable way?** | **3. exposure accurately measured to minimise bias?** | **4. outcome accurately measured to minimise bias?** | **5a. identified all important confounding factors?** | **5b. taken account o f confounding in design and analysis?** | **6. follow up of subjects complete enough?** | **7. results clearly communicated?** | **8. results appear precise?** | **9. results are believeable?** | **10. results can be applied to local population?** | **11. results fit with other available evidence?** | **12. implications of the study clear?** |
| --- | --- | --- | --- | --- | --- | --- | --- | --- | --- | --- | --- | --- | --- | --- |
| Guha, K. and Allen, C. J. and Chawla, S. and Pryse-Hawkins, H. and Fallon, L. and Chambers, V. and Vazir, A. and Lyon, A. R. and Cowie, M. R. and Sharma, R. | Audit of a tertiary heart failure outpatient service to assess compliance with NICE guidelines | YES | YES | YES | YES | CAN'T TELL | CAN'T TELL | YES | YES | YES | YES | YES | YES | YES |
| Murphy, T. and Waterhouse, D. and James, S. and Casey, C. and Fitzgerald, E. and O'Connell, E. and Watson, C. and Gallagher, J. and Ledwidge, M. and McDonald, K. | A Comparison of Hfref Vs Hfpef's Clinical Workload and Cost in the First Year Following Hospitalisation and Enrollment in a Disease Management Program | YES | YES | YES | YES | CAN'T TELL | CAN'T TELL | YES | YES | YES | YES | YES | YES | YES |
| Hawley, A. and He, J. and Crabtree, A. and Iacovides, S. and Keeling, P. | The impact of an integrated heart failure service in a medium-sized district general hospital | YES | YES | YES | YES | CAN'T TELL | CAN'T TELL | YES | YES | YES | YES | YES | YES | YES |
| Cannata, A. and Badawy, L. and Anyu, A. T. and Samways, J. and Sweeney, M. and Jordan-Rios, A. and Zakeri, R. and Scott, P. A. and Piper, S. and Plymen, C. M. and McDonagh, T. A. and Bromage, D. I. | The prognostic impact of specialist cardiology input in patients admitted for heart failure and normal ejection fraction | YES | YES | CAN'T TELL | YES | CAN'T TELL | CAN'T TELL | YES | YES | YES | YES | YES | NO | YES |
| Tran, P. and Long, T. and Smith, J. and Kuehl, M. and Mahdy, T. and Banerjee, P. | Developing a contemporary community clinic for patients with heart failure with preserved ejection fraction within the current National Health Service model | YES | YES | CAN'T TELL | YES | CAN'T TELL | CAN'T TELL | NO | YES | YES | YES | YES | YES | YES |
| Zheng, A. and Cowan, E. and Philip, L. and Guha, K. and Kalra, P. R. and Morton, G. | Characteristics and outcomes of patients with suspected heart failure and elevated natriuretic peptides referred to a nice-compliant heart failure clinic | YES | YES | YES | YES | CAN'T TELL | CAN'T TELL | YES | YES | YES | YES | YES | YES | YES |
| Dulai, R. and Sheikh, A. S. and Qureshi, A. and Katechia, S. and Peysakhova, Y. and Johns, M. and Mazhar, S. | Prevalence, clinical characteristics and outcomes of HF with preserved versus reduced ejection fraction | YES | YES | YES | YES | CAN'T TELL | CAN'T TELL | YES | YES | YES | YES | YES | YES | YES |
| Garg, P. Dakshi, A. Assadi, H. Swift, AJ. Naveed, U. Fent, G. Lewis, Rogers, D.  Charalampopoulos, A. Al-Mohammad A. | Characterisation of the patients with suspected heart failure: experience from the SHEAF registry | YES | YES | YES | YES | CAN'T TELL | CAN'T TELL | YES | YES | YES | YES | YES | YES | YES |
| Morton, G. Philip, L. Gilpin, T. Chan, PR. Guha, K. Kalra, PR. | Does specialist review for patients with suspected heart failure predict better outcomes? An observational study on the utility of compliance with NICE guidelines | YES | YES | YES | YES | CAN'T TELL | CAN'T TELL | YES | YES | YES | YES | YES | YES | YES |
| Peplow & Rees | An evaluation of a service expansion to include patients with heart failure with preserved ejection fraction | YES | CAN'T TELL | CAN'T TELL | YES | CAN'T TELL | CAN'T TELL | YES | YES | YES | YES | YES | CAN'T TELL | CAN'T TELL |
| Tavares et al. | Characteristics and outcomes of patients with heart failure with preserved ejection fraction referred to a community specialist nurse-led clinic | YES | YES | YES | YES | CAN'T TELL | CAN'T TELL | YES | YES | YES | YES | YES | YES | YES |
| Migas et al | Missed opportunities in heart failure diagnosis and management: study of an urban UK population | YES | YES | CAN'T TELL | YES | CAN'T TELL | CAN'T TELL | YES | YES | YES | YES | YES | YES | YES |

## Table 8: Additional quotes in support of themes

| **Theme** | **Quotations in support of Theme** |
| --- | --- |
| **Theme 1. Variability in services and management** | “Patients newly diagnosed with HFpEF have a poor short­-term prognosis with high rates of hospitalizations yet lack access to the MDT” Bolam *et al.*  “There appears to be large variability in follow- up practice, with some electing to follow up all HFPEF patients at least once after the first diagnosis, while others discharge them back to general practitioners with advice.”  “The majority of these patients was elderly (68.9%). 38.8% of patients who received echocardiography were referred for specialist clinic follow-up, with HFrEF patients more likely to be seen in this setting.” Monteiro et al  “In our institution, patients with HFREF are referred to the community heart failure team for further management, whereas, as yet, HFPEF patients are not” Dulai *et al.*  “Those with HFpEF and those without HF (NHF) were usually discharged to primary care with a diagnosis and management plan where possible.” Zheng *et al.* |
| **Theme 2: Uncertainty cardiology led/based care is optimal:** | “So far specialist management of patients may not always be required and further studies are warranted to investigate the role of specialist cardiology care in this setting” Cannata *et al.*  “This timely study has demonstrated that existing general and emerging HFpEF clinics may not comprehensively address the multifaceted aspects of HFpEF as clinic activities concentrated primarily on cardiological measures.” Tran *et al.*  “Our study showed the greatest impact on patients with HFpEF, both with reduced early mortality, and HF readmissions. Benefits were greatest in patients managed on a cardiology ward with specialist HF input.” Hawley *et al.* |
| **Theme 3. Complexity makes management challenging:** | “Our data conﬁrmed that the majority of HF patients have signiﬁcant co-morbidities with 95% of our cohort having moderate or severe CCI.” Nyjo *et al.*  “Our data conﬁrmed that the majority of HF patients have signiﬁcant co-morbidities with 95% of our cohort having moderate or severe CCI.” Migas *et al.*  “Frailty and HFpEF are often intertwined in elderly patients, accompanied by sarcopenia, metabolic dysregulation and deconditioning. These factors generally make them unsuitable for further complicated investigations and complex medication regimens (i.e., polypharmacy). For these reasons, recognising frailty should form an integral part of initial clinic assessments” Tran *et al.*  “Consistent with this, there were a greater number of multidisciplinary team or consultant reviews in the heart failure with preserved ejection fraction cohort, suggesting that they are the more complex heart failure type when it comes to management, and are more likely to require time-consuming reviews. This may be exacerbated by the lack of treatment guidance for this group.” Peplow & Rees  “The comparable long-term event rate between HFnEF patients who received in-hospital specialist cardiology input and those who not may relate to the paucity of guideline-recommended treatments in this cohort of patients” Cannata *et al.*  “We recognize the eminence of HFpEF as the most frequent diagnosis, with its challenges of poor prognosis and paucity of therapeutic options.” Garg *et al.* |
| **Theme 4. Need for service re-design:** | “Despite the recognized limitations of a retrospective study design, this study highlights the need for a renewed focus on HF patient pathways, especially for those with HFpEF.” Migas *et al.*  “As HFpEF becomes the dominant form of community HF, more work is required in HF-DMPs to address prevention of non-cardiovascular re-hospitalizations and to integrate hospital-based HF-DMPs into primary care healthcare structures” Murphy *et al.*  “The current system is not prepared to manage patients with multimorbidity” Tavares *et al.* |

## References

1. Guha K, Allen CJ, Chawla S*, et al.* Audit of a tertiary heart failure outpatient service to assess compliance with NICE guidelines. *Clin Med (Lond)* 2016;**16**:407-411. doi: 10.7861/clinmedicine.16-5-407

2. Murphy TM, Waterhouse DF, James S*, et al.* A comparison of HFrEF vs HFpEF's clinical workload and cost in the first year following hospitalization and enrollment in a disease management program. *Int J Cardiol* 2017;**232**:330-335. doi: 10.1016/j.ijcard.2016.12.057

3. Hassan S, Lee C, Beleznai T*, et al.* P276 Heart failure specialist nurse-led day case ambulatory management with intravenous diuretics reduces hospitalisations for acute decompensated heart failure irrespective of ejection fraction. *European Heart Journal* 2018;**39**:ehy564.P276. doi: 10.1093/eurheartj/ehy564.P276

4. Hawley A, He J, Crabtree A, Iacovides S, Keeling P. The impact of an integrated heart failure service in a medium-sized district general hospital. *Open Heart* 2020;**7**. doi: 10.1136/openhrt-2019-001218

5. Monteiro C, Cojoianu A, Savage R*, et al.* P214 Clinical audit of in-patient echocardiography in acute heart failure: real world data from a tertiary hospital. *European Heart Journal - Cardiovascular Imaging* 2020;**21**. doi: 10.1093/ehjci/jez319.081

6. Cannata A, Badawy L, Anyu AT*, et al.* The prognostic impact of specialist cardiology input in patients admitted for heart failure and normal ejection fraction. *ESC Heart Fail* 2023;**10**:2648-2655. doi: 10.1002/ehf2.14440

7. Simms J, Irani T, Schiff R. 38HEART FAILURE IN THE OLDER PERSON: IS THERE STILL A PLACE FOR THE GERIATRICIAN? *Age and Ageing* 2014;**43**:i9-i9. doi: 10.1093/ageing/afu036.38

8. Tran P, Long T, Smith J*, et al.* Developing a contemporary community clinic for patients with heart failure with preserved ejection fraction within the current National Health Service model. *Open Heart* 2022;**9**. doi: 10.1136/openhrt-2022-002101

9. Shah J, Paz E, Elias E*, et al.* 114 The challenges and opportunities of starting a heart failure virtual ward, experience from london north west university healthcare nhs trust. *Heart* 2022;**108**:A85-A85. doi: 10.1136/heartjnl-2022-BCS.114

10. Thompson A, Crilley J, Wilson D*, et al.* 24 An Epidemic of HFPEF? *Heart* 2016;**102**:A15-A16. doi: 10.1136/heartjnl-2016-309890.24

11. Nguyen M, Rumjaun S, Lowe-Jones R*, et al.* Management and outcomes of heart failure patients with CKD: experience from an inter-disciplinary clinic. *ESC Heart Fail* 2020;**7**:3225-3230. doi: 10.1002/ehf2.12796

12. Sinclair H, Ackrill M, Holdsworth H*, et al.* 88 Rapid access heart failure clinic: impact of a physiologist-delivered service in a uk district general hospital. *Heart* 2019;**105**:A74-A74. doi: 10.1136/heartjnl-2019-BCS.86

13. Zheng A, Cowan E, Mach L*, et al.* Characteristics and outcomes of patients with suspected heart failure referred in line with National Institute for Health and Care Excellence guidance. *Heart* 2020;**106**:1579-1585. doi: 10.1136/heartjnl-2019-316511

14. Horan C, Bower C, Kennedy J*, et al.* Outcomes of heart failure specialist nurse delivered consultant led community virtual heart failure multidisciplinary team meetings during the first peak of the COVID-19 pandemic. 2021. doi: <https://esc365.escardio.org/Presentation/233352/abstract> (Accesses 01/10/2024)

15. Doleman F, Santon M, Clewes J*, et al.* P2593Evaluation of an ambulatory heart failure service - a 2 years' experience. *European Heart Journal* 2019;**40**. doi: 10.1093/eurheartj/ehz748.0919

16. Al-Mohammad A, Watt V, O'Toole L, Hall I, Yates L. Insights into the epidemiology of incident Heart Failure (HF): outcomes of rapid HF access clinic applying the NICE guidelines. *European Heart Journal* 2013;**34**. doi: 10.1093/eurheartj/eht309.P4225

17. Dulai R, Sheikh AS, Qureshi A*, et al.* Prevalence, clinical characteristics and outcomes of HF with preserved versus reduced ejection fraction. *Br J Cardiol* 2016;**23**:1-40. doi:

18. Bolam H, Kalra P, Guha K, Morton D. Abstract: P2005 The impact of a clinical educational and self-care intervention in patients with heart failure with preserved ejection fraction. *European Journal of Heart Failure.* 2019. doi:

19. Griffiths A, Taylor J, McRae D. Evaluation of a specialist pharmacist led diagnostic chronic heart failure clinic. 2020. doi:

20. Garg P, Dakshi A, Assadi H*, et al.* Characterisation of the patients with suspected heart failure: experience from the SHEAF registry. *Open Heart* 2021;**8**. doi: 10.1136/openhrt-2020-001448

21. Morton G, Philip L, Gilpin T*, et al.* Does specialist review for patients with suspected heart failure predict better outcomes? An observational study on the utility of compliance with NICE guidelines. *BMJ Open* 2018;**8**:e021856. doi: 10.1136/bmjopen-2018-021856

22. Peplow J, Rees S. An evaluation of a service expansion to include patients with heart failure with preserved ejection fraction. *British Journal of Cardiac Nursing* 2024;**19**:1-10. doi: 10.12968/bjca.2023.0082

23. Tavares S, Kanaganayagam G, Lampridou S*, et al.* Characteristics and outcomes of patients with heart failure with preserved ejection fraction referred to a community specialist nurse-led clinic. *British Journal of Cardiac Nursing* 2024;**19**:1-14. doi: 10.12968/bjca.2024.0012

24. Migas S, Ellis ML, Wrona B*, et al.* Missed opportunities in heart failure diagnosis and management: study of an urban UK population. *ESC Heart Failure* 2024;**11**:2200-2213. doi: <https://doi.org/10.1002/ehf2.14766>

25. Murphy N, Duvva D, Kelly A-M*, et al.* 168 Can the use of a structured management approach using abcdef mnemonic to manage multi-morbidity in HFpEF improve clinical outcomes? *Heart* 2024;**110**:178-178. doi: 10.1136/heartjnl-2024-BCS.165

26. 28th Annual Meeting of the European Association of Cardiothoracic Anaesthesiologists, EACTA 2013. *Applied cardiopulmonary pathophysiology* 2014;**17**:233‐234. doi:

27. Partners. KH. Heart Failure Specialist Nurse Service Operational Procedures Acute and Community Setting. 2017. doi: Not publically available, please contact the healthcare service to obtain

28. Healthcare. IC. Chronic Heart Failure Guide. doi: Not publically available, please contact the healthcare service to obtain

29. Partners. SHaC. Heart Failure Diagnostic and Treatment Pathway in Primary Care. 2021. doi: Not publically available, please contact the healthcare service to obtain

30. Hospitals. OU. Heart Failure Guidance Summary for GPs. 2021. doi: Not publically available, please contact the healthcare service to obtain

31. Midlands. UHoN. Standard Operating Procedure for the Ambulatory Heart Failure Units. 2020. doi: Not publically available, please contact the healthcare service to obtain

32. NHS AaA. Developing a pathway for individuals with HFpEF - Project Evaluation. 2024. doi: Not publically available, please contact the healthcare service to obtain

33. Care. WHa. Standard Operational Procedure - Community Heart Failure Service Ops. 2024. doi: Not publically available, please contact the healthcare service to obtain

34. Partnership L. Standard operating procudre - Heart Failure Specialist Nursing Service Leicestershire Partnership NHS Trust. 2024. doi: Not publically available, please contact the healthcare service to obtain

35. Trust. WHN. West Hertforshire proposed model for heart failure nurse service West Herts. 2019. doi: Not publically available, please contact the healthcare service to obtain
